# An Information Theoretic Interpretation to Deep Neural Networks †

**DOI:** 10.3390/e24010135

**Published:** 2022-01-17

**Authors:** Xiangxiang Xu, Shao-Lun Huang, Lizhong Zheng, Gregory W. Wornell

**Affiliations:** 1 Data Science and Information Technology Research Center, Tsinghua–Berkeley Shenzhen Institute, Shenzhen 518055, China; xuxx@mit.edu; 2 Department of Electrical Engineering and Computer Science, Massachusetts Institute of Technology, Cambridge, MA 02139, USA; lizhong@mit.edu (L.Z.); gww@mit.edu (G.W.W.)

**Keywords:** deep neural network, information theory, local information geometry, feature extraction

## Abstract

With the unprecedented performance achieved by deep learning, it is commonly believed that deep neural networks (DNNs) attempt to extract informative features for learning tasks. To formalize this intuition, we apply the local information geometric analysis and establish an information-theoretic framework for feature selection, which demonstrates the information-theoretic optimality of DNN features. Moreover, we conduct a quantitative analysis to characterize the impact of network structure on the feature extraction process of DNNs. Our investigation naturally leads to a performance metric for evaluating the effectiveness of extracted features, called the H-score, which illustrates the connection between the practical training process of DNNs and the information-theoretic framework. Finally, we validate our theoretical results by experimental designs on synthesized data and the ImageNet dataset.

## 1. Introduction

Due to the striking performance of deep learning in various application fields, deep neural networks (DNNs) have gained great attention in modern computer science. While it is a common understanding that the features extracted from the hidden layers of DNN are “informative” for learning tasks, the mathematical meaning of informative features in DNN is generally not clear. From the practical perspective, DNN models have obtained unprecedented performance in varying tasks, such as image recognition [1], language processing [2,3], and games [4,5]. However, the understanding of the feature extraction behind these models is relatively lacking, which poses challenges for their application in security-sensitive tasks, such as the autonomous vehicle.

To address this problem, there have been numerous research efforts, including both experimental and theoretical studies [6]. The experimental studies usually focus on some empirical properties of the feature extracted by DNNs, by visualizing the feature [7] or testing its performance on specific training settings [8] or learning tasks [9]. Though such empirical methods have provided some intuitive interpretations, the performance can highly depend on the data and network architecture used. For example, while the feature visualization works well on convolutional neural networks, its application to other networks is typically less effective [10].

In contrast, theoretical studies focus on the analytical properties of the extracted feature or the learning process in DNNs. Due to the complicated structure of DNNs, existing studies were often restricted to the networks of specific structures, e.g., network with infinite width [11] or two-layer network [12,13], to characterize the theoretical behaviors. However, the interpretation of the optimal feature remains unclear, which limits their further applications. To obtain better interpretability, tools and measures from information theory [14] have recently been applied to connect DNNs with general information processing problems [15]. For instance, the information bottleneck [16,17] employs the mutual information as the metric to quantify the informativeness of features in DNN, and other information metrics, such as the Kullback–Leibler (KL) divergence [18] and Weissenstein distance [19], are also used in different problems. However, there is still a disconnection between these information metrics and the performance objectives of the inference tasks that DNNs want to solve [20]. Therefore, it is, in general, difficult to match the DNN learning with the optimization of a particular information metric.

This paper aims to provide an information-theoretic interpretation to the feature extraction process in DNNs, to bridge the gap between the practical deep learning implementations and information-theoretic characterizations. To this end, we first propose an information-theoretic feature selection framework, which establishes an information metric to measure the performance of each given feature in inference tasks. In addition, we demonstrate that the optimal features extracted by DNNs coincide with the solutions of the information-theoretic feature selection problem, which share the same performance metric. Therefore, our results give an explicit interpretation of the learning goal of the back-propagation (BackProp) and stochastic gradient descent (SGD) operations in deep learning [21], which also lead to a performance metric for evaluating the effectiveness of the extracted features. Finally, we validate our theoretic characterizations using numerical experiments on both synthesized data and the ImageNet [22] dataset for image classification.

## 2. Preliminaries and Methods

### 2.1. Methodological Background

The main method used in our development is local information geometry [23,24], which characterizes the local geometric properties of the probability distribution space. The local information geometric method is closely related to the conventional Hirschfeld–Gebelein–Rényi (HGR) maximal correlation [25,26,27] problem, which has attracted increasing interest in the information theory community [28,29,30,31,32,33], and has also been applied in data analysis [34] and privacy studies [35].

Specifically, we use the local information geometric method to construct and investigate an information-theoretic feature selection problem in Section 3.1, which leads to an information metric of features and also demonstrates an SVD (singular value decomposition) structure of the feature selection process. Following the same analysis framework, we characterize the optimal feature extracted by DNNs in Section 3.2, and demonstrate that the same SVD structure is shared by DNNs. Based on the established connection, we then propose an effectiveness measure for DNNs, with details presented in Section 3.3.

### 2.2. Notations

Throughout this paper, we use *X*, X, $P_{X}$, and *x* to represent a discrete random variable, the range, the probability distribution, and the value of *X*. In addition, for any function $s\left(X\right)\in R^{k}$ of *X*, we use $\mu_{s}$ to denote the mean of s(X), and “$\tilde{\;}$” to denote the centered variable with mean subtracted, e.g., $\tilde{s}\left(X\right)≜s\left(X\right)-\mu_{s}$. Moreover, we use ∥·∥ and ${∥\cdot ∥}_{F}$ to denote the $\ell_{2}$-norm and the Frobenius norm, respectively. All logarithms in our analyses are base *e*, i.e., natural.

### 2.3. Local Information Geometry

The following concepts from local information geometry would be useful in our development.

**Definition** **1** (ϵ-Neighborhood). *Let $P^{X}$ denote the space of distributions on some finite alphabet X, and let $\mathrm{relint}(P^{X})$ denote the subset of strictly positive distributions. For a given ϵ>0, the ϵ-neighborhood of a distribution $P_{X}\in \mathrm{relint}\left(P^{X}\right)$ is defined by the $\chi^{2}$-divergence as*

$$N_{\epsilon }^{X}\left(P_{X}\right)≜\left\{P\in P^{X}:\sum_{x\in X}\frac{{\left(P\left(x\right)-P_{X}\left(x\right)\right)}^{2}}{P_{X}\left(x\right)}\leq \epsilon^{2}\right\}.$$

**Definition** **2** (ϵ-Dependence). *The random variables X,Y are called ϵ-dependent if $P_{XY}\in N_{\epsilon }^{X\times Y}\left(P_{X}P_{Y}\right)$.*

**Definition** **3** (ϵ-Attribute). *A random variable U is called an ϵ-attribute of X if $P_{X|U}\left(\cdot |u\right)\in N_{\epsilon }^{X}\left(P_{X}\right)$, for all u∈U.*

We will focus on the small ϵ regime, which we refer to as the *local analysis regime*. In addition, for any $P\in P^{X}$, we define the *information vector*ϕ and *feature function*L(x) corresponding to *P*, with respect to a reference distribution $P_{X}\in \mathrm{relint}\left(P^{X}\right)$, as

(1) $$\phi \left(x\right)≜\frac{P\left(x\right)-P_{X}\left(x\right)}{\sqrt{P_{X}\left(x\right)}},\;L\left(x\right)≜\frac{\phi (x)}{\sqrt{P_{X}\left(x\right)}}.$$

This gives a three way correspondence P↔ϕ↔L for all distributions in $N_{\epsilon }^{X}\left(P_{X}\right)$, which will be useful in our derivations.

### 2.4. Modal Decomposition

Given a pair of discrete random variables X,Y with the joint distribution $P_{XY}\left(x,y\right)$, the |Y|×|X| matrix $\tilde{B}$ is defined as

(2) $$\tilde{B}\left(y,x\right)≜\frac{P_{XY}\left(x,y\right)-P_{X}\left(x\right)P_{Y}\left(y\right)}{\sqrt{P_{X}\left(x\right)P_{Y}\left(y\right)}},$$

where $\tilde{B}\left(y,x\right)$ is the (y,x)th entry of $\tilde{B}$. The matrix $\tilde{B}$ is referred to as the canonical dependence matrix (CDM) [24]. The SVD of $\tilde{B}$ is referred to as the *modal decomposition* [24] of the joint distribution $P_{XY}$, which has the following property [18].

**Lemma** **1.** *The SVD of $\tilde{B}$ can be written as $\tilde{B}=\sum_{i=1}^{K}\sigma_{i}\;\psi_{i}^{Y}{\left(\psi_{i}^{X}\right)}^{T}$, where K≜min{|X|,|Y|}, and $\sigma_{i}$ denotes the ith singular value with the ordering $1\geq \sigma_{1}\geq \ldots \geq \sigma_{K}=0$, and $\psi_{i}^{Y}$ and $\psi_{i}^{X}$ are the corresponding left and right singular vectors with $\psi_{K}^{X}\left(x\right)=\sqrt{P_{X}\left(x\right)}$ and $\psi_{K}^{Y}\left(y\right)=\sqrt{P_{Y}\left(y\right)}$.*

This SVD decomposes the feature spaces of X,Y into maximally correlated features. To see that, consider the generalized canonical correlation analysis (CCA) problem:

(3) $$\begin{array}{c} \max_{\begin{array}{c} E\left[f_{i}\left(X\right)\right]=E\left[g_{i}\left(Y\right)\right]=0\\ E\left[f_{i}\left(X\right)\;f_{j}\left(X\right)\right]=E\left[g_{i}\left(Y\right)\;g_{j}\left(Y\right)\right]=\delta_{ij} \end{array}}\sum_{i=1}^{k}E\left[f_{i}\left(X\right)\;g_{i}\left(Y\right)\right], \end{array}$$

where $\delta_{ij}$ denotes the Kronecker delta function. It can be shown that for any 1≤k≤K−1, the optimal features are $f_{i}\left(x\right)=\psi_{i}^{X}\left(x\right)/\sqrt{P_{X}\left(x\right)}$, and $g_{i}\left(y\right)=\psi_{i}^{Y}\left(y\right)/\sqrt{P_{Y}\left(y\right)}$, for i=0,…,K−1, where $\psi_{i}^{X}\left(x\right)$ and $\psi_{i}^{Y}\left(y\right)$ are the *x*th and *y*th entries of $\psi_{i}^{X}$ and $\psi_{i}^{Y}$, respectively [18]. The special case k=1 corresponds to the HGR maximal correlation [25,26,27], and the optimal features can be computed from the ACE (Alternating Conditional Expectation) algorithm [36].

### 2.5. Deep Neural Networks

**Figure 1:**
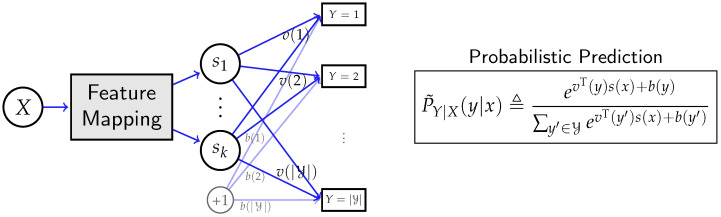
A deep neural network that uses data *X* to predict *Y*. All hidden layers together map the input data *X* to *k*-dimensional feature $s\left(x\right)={\left(s_{1},\ldots ,s_{k}\right)}^{T}$. Then, the probabilistic prediction ${\tilde{P}}_{Y|X}$ of *Y* is computed from s(x),v(y), and b(y), where *v* and bias *b* are the weights and bias in the last layer.

The architecture of deep neural networks (under log-loss) can be depicted as Figure 1, where *X* is the input data, e.g., images, audios, or natural languages. Moreover, *Y* is the objective to predict, which can represent a discrete label in classification tasks, or represent target natural languages in machine translations [37]. Specifically, for given data *X*, the network produces a (trainable) feature mapping to generate *k*-dimensional feature $s\left(x\right)={\left(s_{1},\ldots ,s_{k}\right)}^{T}$. In practice, the feature mapping block (depicted as the gray block in Figure 1) is typically composed of hundreds and thousands of functional components (e.g., residual block [1]) with different types of layers, and may contain recurrent structure, e.g., LSTM (Long Short-Term Memory) [38]. In general, the internal structure of the feature mapping can have various different types of designs, depending on the learning tasks.

After obtaining the feature s(X), the *Y* is then predicted by the probability distribution ${\tilde{P}}_{Y|X}^{\left(s,v,b\right)}$ of the form

(4) $$\begin{array}{c} {\tilde{P}}_{Y|X}^{\left(s,v,b\right)}\left(y|x\right)≜\frac{e^{v^{T}\left(y\right)s\left(x\right)+b\left(y\right)}}{\sum_{y^{\prime }\in Y}e^{v^{T}\left(y^{\prime }\right)s\left(x\right)+b\left(y^{\prime }\right)}}, \end{array}$$

which is obtained by applying the softmax function [39] on $v^{T}\left(y\right)s\left(x\right)+b\left(y\right)$, where v(·) and b(·) are the weights and biases in the last layer, respectively (this is equivalent to the common practice that denotes weight and biases by the matrix ${\left[v\left(1\right),\ldots ,v(|Y|)\right]}^{T}$ and the vector ${\left[b\left(1\right),\ldots ,b(|Y|)\right]}^{T}$, respectively. However, as we will show later, expressing weights *v* and biases *b* as mappings of *y* can better illustrate their roles in feature selection). We will use ${\tilde{P}}_{Y|X}$ to refer to ${\tilde{P}}_{Y|X}^{\left(s,v,b\right)}$ when there is no ambiguity.

Then, for a given training set of labeled samples $\left(x_{i},y_{i}\right)$, for i=1,…,N, all the parameters in the network, including *v*, *b*, as well as those in the feature mapping block, are chosen to maximize the log-likelihood function (or, equivalently, minimize the log-loss)

(5) $$\begin{array}{c} \frac{1}{N}\sum_{i=1}^{N}\log{\tilde{P}}_{Y|X}\left(y_{i}|x_{i}\right). \end{array}$$

The procedure of choosing such parameters is called the training of network, which can be performed by stochastic gradient descent (SGD) or its variants [21]. With a trained network, the label $\hat{y}$ for a new data sample *x* can be predicted by the maximum a posteriori (MAP) estimation, i.e., $\hat{y}\;=\;{arg max}_{y\in Y}{\tilde{P}}_{Y|X}\left(y|x\right).$ Specifically, when we make predictions for samples in a test dataset, the proportion of samples with correct prediction (i.e., $\hat{y}=y$) over all samples is called the test accuracy.

## 3. Results

### 3.1. Information-Theoretic Feature Selection

Suppose that, given random variables X,Y with joint distribution $P_{XY}$, we want to infer about an attribute *V* of *Y* from observed i.i.d. samples $x_{1},\ldots ,x_{n}$ of *X*. When the statistical model $P_{X|V}$ is known, the optimal decision rule is the log-likelihood ratio test, where the log-likelihood function can be viewed as the optimal feature for inference. However, in many practical situations [18], it is hard to identify the model of the targeted attribute, and it is necessary to select low-dimensional informative features of *X* for inference tasks before knowing the model. An information-theoretic formulation of such feature selection problem is the universal feature selection problem [24], which we formalize as follows.

To begin, for an attribute *V*, we refer to $C_{Y}=\left\{\;V,\;\left\{P_{V}\left(v\right),\;v\in V\right\},\;\left\{\phi_{v}^{Y|V},\;v\in V\right\}\right\},$ as the *configuration* of *V*, where $\phi_{v}^{Y|V}↔P_{Y|V}\left(\cdot |v\right)$ is the information vector specifying the corresponding conditional distribution $P_{Y|V}\left(\cdot |v\right)$. The configuration of *V* models the statistical correlation between *V* and *Y*. In the sequel, we focus on the local analysis regime, for which we assume that all the attributes *V* of our interests to detect are ϵ-attributes of *Y*. As a result, the corresponding configuration satisfies $∥\phi_{v}^{Y|V}∥\leq \epsilon$, for all v∈V. We refer to such configurations as *ϵ-configurations*. The configuration of *V* is unknown in advance but assumed to be generated from a *rotational invariant ensemble (RIE)*.

**Definition** **4** (RIE). *Two configurations $C_{Y}$ and ${\tilde{C}}_{Y}$ defined as*

$$C_{Y}≜\left\{\;V,\;\left\{P_{V}\left(v\right),\;v\in V\right\},\;\left\{\phi_{v}^{Y|V},\;v\in V\right\}\right\},$$

C˜Y≜V,{PV(v),v∈V},{ϕ˜ϕvY|V,v∈V}

*are called rotationally equivalent, if there exists a unitary matrix Q such that ϕ˜ϕvY|V=QϕvY|V, for all v∈V. Moreover, a probability measure defined on a set of configurations is called an RIE, if all rotationally equivalent configurations have the same measure.*

The RIE can be interpreted as assigning a uniform measure to the attributes with the same level of distinguishability. To infer about the attribute *V*, we construct a *k*-dimensional feature vector $h^{k}=\left(h_{1},\ldots ,h_{k}\right)$, for some 1≤k≤K−1, of the form

(6) $$\begin{array}{c} h_{i}=\frac{1}{n}\sum_{l=1}^{n}f_{i}\left(x_{l}\right),\;i=1,\ldots ,k, \end{array}$$

for some choices of feature functions $f_{i}$. Our goal is to determine the $f_{i}$ such that the optimal decision rule based on $h^{k}$ achieves the smallest possible error probability, where the performance is averaged over the possible $C_{Y}$ generated from an RIE. In turn, we denote $\xi_{i}^{X}↔f_{i}$ as the corresponding information vector, and define the matrix $\Xi^{X}≜\left[\xi_{1}^{X}\;\ldots \;\xi_{k}^{X}\right]$.

**Theorem** **1** (Universal Feature Selection). *For $v,v^{\prime }\in V$, let $E_{h^{k}}\left(v,v^{\prime }\right)$ be the error exponent associated with the pairwise error probability distinguishing v and $v^{\prime }$ based on $h^{k}$, then the expected error exponent over a given RIE defined on the set of ϵ-configurations is given by*

(7) $$\begin{array}{cc} E\left[E_{h^{k}}\left(v,v^{\prime }\right)\right]\; & =\frac{C_{0}}{2}\cdot {∥\tilde{B}\Xi^{X}{\left({\left(\Xi^{X}\right)}^{T}\Xi^{X}\right)}^{-\frac{1}{2}}∥}_{F}^{2}+o\left(\epsilon^{2}\right), \end{array}$$

*where $C_{0}≜\frac{1}{4|Y|}\cdot E\left[{∥\phi_{v}^{Y|V}-\phi_{v^{\prime }}^{Y|V}∥}^{2}\right]$ is independent of the choices of $f_{i}$’s, and the expectations $E\left[\cdot \right]$ are taken over this RIE.*

**Proof.**  See Appendix A. □

As a result of (7), designing the $\xi_{i}^{X}$ as the singular vectors $\psi_{i}^{X}$ of $\tilde{B}$, for i=1,…,k, optimizes (7) for all RIEs, pairs of $\left(v,v^{\prime }\right)$, and ϵ-configurations. Thus, the feature functions corresponding to $\psi_{i}^{X}$ are *universally optimal* for inferring the unknown attribute *V*. Moreover, (7) naturally leads to an information metric ${∥\tilde{B}\Xi^{X}{\left({\left(\Xi^{X}\right)}^{T}\Xi^{X}\right)}^{-\frac{1}{2}}∥}_{F}^{2}$ for any feature $\Xi^{X}$ of *X*, measured by projecting the normalized $\Xi^{X}$ through a linear projection $\tilde{B}$. This information metric quantifies how informative a feature of *X* is when solving inference problems with respect to *Y* and is optimized when designing features by singular vectors of $\tilde{B}$. Thus, we can interpret the universal feature selection as solving the most informative features for data inferences via the SVD of $\tilde{B}$, which also coincides with the maximally correlated features in (3). Later, we will show that the feature selection in DNNs shares the same information metric as universal feature selection in the local analysis regime.

### 3.2. Feature Extraction in Deep Neural Networks

#### 3.2.1. Network with Ideal Expressive Power

For convenience of analysis, we first consider the ideal case where the neural network can express any feature mapping s(·) as desired. While this assumption can be rather strong, the existence of such ideal networks is guaranteed by the universal approximation theorem [40]. In addition, one goal of practical network designs is to approximate the ideal networks and obtain sufficient expressive power. For such networks, we will show that when X,Y are ϵ-dependent, the extracted feature s(x) and weights v(y) coincide with the solutions of the universal feature selection.

To begin, we use $P_{XY}$ to denote the joint empirical distribution of the labeled samples $(x_{i},y_{i}),i=1,\ldots ,N$, and $P_{X},P_{Y}$ to denote the corresponding marginal distributions. Then, the objective function of (5) is the empirical average of the log-likelihood function

$$\begin{array}{c} \frac{1}{N}\sum_{i=1}^{N}\log{\tilde{P}}_{Y|X}\left(y_{i}|x_{i}\right)=E_{P_{XY}}\left[\log{\tilde{P}}_{Y|X}\left(Y|X\right)\right]. \end{array}$$

Therefore, maximizing this empirical average is equivalent as minimizing the KL divergence:

(8) $$\begin{array}{c} \left(s^{*},v^{*},b^{*}\right)=\underset{\left(s,v,b\right)}{arg min}\;D\left(P_{XY}∥P_{X}\;{\tilde{P}}_{Y|X}^{\left(s,v,b\right)}\right). \end{array}$$

This can be interpreted as finding the best fitting to empirical joint distribution $P_{XY}$ by distributions of the form $P_{X}\;{\tilde{P}}_{Y|X}^{\left(s,v,b\right)}$. In our development, it is more convenient to denote the bias by $d\left(y\right)=b\left(y\right)-\log P_{Y}\left(y\right)$, for y∈Y. Then, the following lemma illustrates the explicit constraint on the problem (8) in the local analysis regime.

**Lemma** **2.** *If X,Y are ϵ-dependent, then the optimal v,d for (8) satisfy*

(9) $$\begin{array}{c} |{\tilde{v}}^{T}\left(y\right)s\left(x\right)+\tilde{d}\left(y\right)|=O\left(\epsilon \right),\;for\;all\;x\in X,\;y\in Y. \end{array}$$

**Proof.**  See Appendix B. □

In turn, we take (9) as the constraint for solving the problem (8) in the local analysis regime. Moreover, we define the information vectors for zero-mean vectors $\tilde{s}$, $\tilde{v}$ as $\xi^{X}\left(x\right)=\sqrt{P_{X}\left(x\right)}\;\tilde{s}\left(x\right)$, $\xi^{Y}\left(y\right)=\sqrt{P_{Y}\left(y\right)}\;\tilde{v}\left(y\right)$, and define matrices

$$\begin{array}{c} \Xi^{Y}≜{\left[\begin{array}{c} \begin{array}{ccc} \xi^{Y}\left(1\right) & \cdots & \xi^{Y}\left(|Y|\right) \end{array} \end{array}\right]}^{T},\;\Xi^{X}≜{\left[\begin{array}{ccc} \xi^{X}\left(1\right) & \cdots & \xi^{X}\left(|X|\right) \end{array}\right]}^{T}. \end{array}$$

**Lemma** **3.** *The KL divergence (8) in the local analysis regime (9) can be expressed as*

(10) $$\begin{array}{cc} D(P_{XY}∥P_{X}\;{\tilde{P}}_{Y|X}^{\left(s,v,b\right)})\; & =\frac{1}{2}{∥\tilde{B}-\Xi^{Y}{\left(\Xi^{X}\right)}^{T}∥}_{F}^{2}+\frac{1}{2}\eta^{\left(v,b\right)}\left(s\right)+o\left(\epsilon^{2}\right), \end{array}$$

*where $\eta^{\left(v,b\right)}\left(s\right)≜E_{P_{Y}}\left[{\left(\mu_{s}^{T}\tilde{v}\left(Y\right)+\tilde{d}\left(Y\right)\right)}^{2}\right]$.*

**Proof.**  See Appendix C. □

Lemma 3 reveals key insights for feature selection in neural networks. To see this, we consider the following two learning problems: learning the optimal weight *v* for given *s* and learning the optimal feature *s* for given *v*.

For the case that *s* is fixed, we can optimize (10) with $\Xi^{X}$ fixed and obtain the following optimal weights:

**Theorem** **2.** For fixed $\Xi^{X}$ and $\mu_{s}$, the optimal $\Xi^{Y}{}^{*}$ to minimize (10) is given by

(11) $$\begin{array}{c} \Xi^{Y}{}^{*}=\tilde{B}\;\Xi^{X}{\left({\left(\Xi^{X}\right)}^{T}\Xi^{X}\right)}^{-1}, \end{array}$$

and the optimal weights ${\tilde{v}}^{*}$ and bias ${\tilde{d}}^{*}$ are

(12) $$\begin{array}{c} {\tilde{v}}^{*}\left(y\right)=E_{P_{X|Y}}\left[\Lambda_{\tilde{s}\left(X\right)}^{-1}\;\tilde{s}\left(X\right)|Y=y\right],\;{\tilde{d}}^{*}\left(y\right)=-\mu_{s}^{T}\;\tilde{v}\left(Y\right). \end{array}$$

*where $\Lambda_{\tilde{s}\left(X\right)}$ denotes the covariance matrix of $\tilde{s}\left(X\right)$.*

**Proof.**  See Appendix D. □

Specifically, when s(x)=x, Theorem 2 gives the optimal weights for softmax regression. Note that Equation (11) can be viewed as a projection of the input feature $\tilde{s}\left(x\right)$, to a feature v(y) computable from the value of *y*, which is the most correlated feature to $\tilde{s}\left(x\right)$. The solution is given by the operation that left multiplies $\tilde{B}$ matrix, which we refer to as *forward feature projection*.

**Remark** **1.** *While we assume the continuous input s(x) is a function of a discrete variable X, we only need the labeled samples between s and Y to compute the weights and bias from the conditional expectation (12), and the correlation between X and s is irrelevant. Thus, our analysis for weights and bias can be applied to continuous input networks by just ignoring X and taking s as the real input to network.*

We then consider the “backward feature projection” problem, which attempts to find informative feature $s^{*}\left(X\right)$ to minimize the loss (10) with given weights and bias. In particular, we can show that the solution of this backward feature projection is precisely symmetric to the forward one.

**Theorem** **3.** For fixed $\Xi^{Y}$ and $\tilde{d}$, the optimal $\Xi^{X}{}^{*}$ to minimize (10) is given by

(13) $$\begin{array}{c} \Xi^{X}{}^{*}={\tilde{B}}^{T}\;\Xi^{Y}((\Xi^{Y}{)}^{T}\Xi^{Y}{)}^{-1}, \end{array}$$

and the optimal feature function $s^{*}$, which are decomposed to ${\tilde{s}}^{*}$ and $\mu_{s}^{*}$, is given by

(14) $$\begin{array}{cc} {\tilde{s}}^{*}\left(x\right) & =E_{P_{Y|X}}\left[\Lambda_{\tilde{v}\left(Y\right)}^{-1}\;\tilde{v}\left(Y\right)|X=x\right],\\ \mu_{s}^{*} & =-\Lambda_{\tilde{v}\left(Y\right)}^{-1}\;E_{P_{Y}}\left[\tilde{v}\left(Y\right)\;\tilde{d}\left(Y\right)\right], \end{array}$$

*where $\Lambda_{\tilde{v}\left(Y\right)}$ denotes the covariance matrix of $\tilde{v}\left(Y\right)$.*

**Proof.**  See Appendix D. □

Finally, when both *s* and (v,b) (and hence $\Xi^{X},\Xi^{Y},d$) can be designed, the optimal $\left(\Xi^{Y},\Xi^{X}\right)$ corresponds to the low rank factorization of $\tilde{B}$, and the solutions coincide with the universal feature selection.

**Theorem** **4.** The optimal solutions for weights and bias to minimize (10) are given by $\tilde{d}\left(y\right)=-\mu_{s}^{T}\tilde{v}\left(y\right)$, and ${\left(\Xi^{Y},\Xi^{X}\right)}^{*}$ chosen as the largest k left and right singular vectors of $\tilde{B}$.

**Proof.**  See Appendix E. □

Therefore, we conclude that the learning of neural networks, when both *s* and (v,b) are designable, is to extract the most correlated aspects of the input data *X* and the label *Y* that are informative features for data inferences from universal feature selection.

In the practical learning process of DNN, the BackProp updates the weights of the softmax layer and those on the previous layer(s) in an iterative manner. As we have illustrated in Lemma 3, such iterative updates will converge to the same solution as the alternating between the forward feature projection (11) and the backward feature projection (13), which is indeed the power method to solve the SVD for $\tilde{B}$ [41], also known as the Alternating Conditional Expectation (ACE) algorithm [36].

**Remark** **2.** *From Theorem 4, for a neural network with sufficient expressive power, the trained feature depends only on the distribution of input data rather than the training process. It is worth mentioning that this result does not contradict the practice that trained weights in hidden layers can be different during each training run. In fact, due to the over-parameterized nature of practical network designs, there exist multiple choices of weights in hidden layers to express the same optimal feature s(x).*

#### 3.2.2. Network with Restricted Expressive Power

The analysis of the previous section has considered neural networks with ideal expressive power, where the feature s(X) can be selected as any desired function. In general, however, the form of feature functions that can be generalized is often limited by the network structure. In the following, we consider networks with restricted expressive power to characterize the impacts of network structure on the extracted feature.

**Figure 2:**
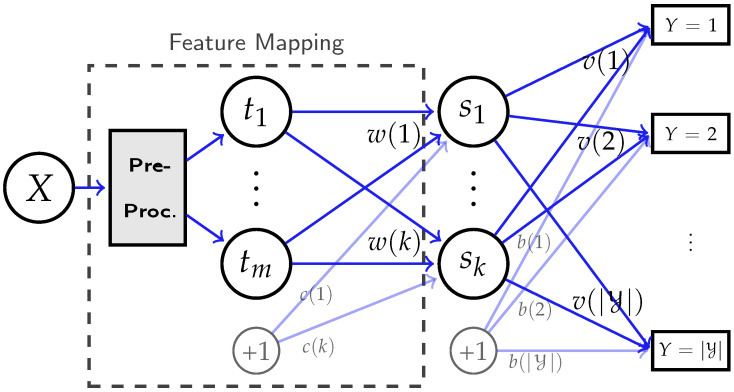
A multi-layer neural network, where the expressive power of the feature mapping s(·) is restricted by the hidden representation *t*. All hidden layers previous to *t* are fixed, represented by the “pre-processing” module.

For illustration, we consider the neural network with a hidden layer of *k* nodes, and a zero-mean continuous input $t={\left[t_{1}\;\cdots \;t_{m}\right]}^{T}\in R^{m}$ to this hidden layer, where *t* is assumed to be a function t(x) of some discrete variable *X*. Our goal is to analyze the weights and bias in this layer with labeled samples $\left(t\left(x_{i}\right),y_{i}\right)$. Assume the activation function of the hidden layer is a generally smooth function σ(·), then the output $s_{z}\left(X\right)$ of the *z*-th hidden node is

(15) $$\begin{array}{c} s_{z}\left(x\right)=\sigma \left(w^{T}\left(z\right)t\left(x\right)+c\left(z\right)\right),\;\mathrm{for}\;z=1,\ldots ,k,\;x\in X, \end{array}$$

where $w\left(z\right)\in R^{m}$ and c(z)∈R are the weights and bias from input layer to hidden layer as shown in Figure 2. We denote $s={\left[s_{1}\;\cdots \;s_{k}\right]}^{T}$ as the input vector to the output classification layer.

To interpret the feature selection in hidden layers, we fix (v(y),b(y)) at the output layer and consider the problem of designing (w(z),c(z)) to minimize the loss function (8) at the output layer. Ideally, we should have picked w(z) and c(z) to generate s(x) to match $s^{*}\left(x\right)$ from (14), which minimizes the loss. However, here we have the constraint that s(x) must take the form of (15) and, intuitively, the network should select w(z),c(z) so that s(x) is close to $s^{*}\left(x\right)$. Our goal is to quantify the notion of such closeness.

To develop insights on feature selection in hidden layers, we again focus on the local analysis regime, where the weights and bias are assumed to satisfy the local constraint

(16) $$\begin{array}{c} \left|{\tilde{v}}^{T}\left(y\right)s\left(x\right)+\tilde{d}\left(y\right)\right|=O\left(\epsilon \right),\;\left|w^{T}\left(z\right)\tilde{t}\left(x\right)\right|=O\left(\epsilon \right),\;\forall x,y,z. \end{array}$$

Then, since *t* is zero-mean, we can express (15) as

(17) $$\begin{array}{c} s_{z}\left(x\right)=\sigma \left(w^{T}\left(z\right)t\left(x\right)+c\left(z\right)\right)=w^{T}\left(z\right)\tilde{t}\left(x\right)\cdot \sigma^{\prime }\left(c(z)\right)+\sigma \left(c(z)\right)+o\left(\epsilon \right), \end{array}$$

Moreover, we define a matrix ${\tilde{B}}_{1}$ with the (z,x)th entry ${\tilde{B}}_{1}\left(z,x\right)=\frac{\sqrt{P_{X}\left(x\right)}}{\sigma^{\prime }\left(c\left(z\right)\right)}{\tilde{s}}_{z}^{*}\left(x\right)$, which can be interpreted as a generalized CDM for the hidden layer. Furthermore, we denote $\xi_{1}^{X}\left(x\right)=\sqrt{P_{X}\left(x\right)}\;\tilde{t}\left(x\right)$ as the information vector of $\tilde{t}\left(x\right)$ with the matrix $\Xi_{1}^{X}$ defined as $\Xi_{1}^{X}≜{\left[\begin{array}{ccc} \xi_{1}^{X}\left(1\right) & \cdots & \xi_{1}^{X}\left(|X|\right) \end{array}\right]}^{T}$, and we also define

(18) $$\begin{array}{c} W≜{\left[\begin{array}{ccc} w(1) & \cdots & w(k) \end{array}\right]}^{T}, \end{array}$$

(19) $$\begin{array}{c} J≜\mathrm{diag}\{\sigma^{\prime }\left(c\left(1\right)\right),\sigma^{\prime }\left(c\left(2\right)\right),\cdots ,\sigma^{\prime }\left(c\left(k\right)\right)\}. \end{array}$$

The following theorem characterizes the loss (8).

**Theorem** **5.** *Given the weights and bias (v,b) at the output layer, and for any input feature s, we denote L(s) as the loss (8) evaluated with respect to (v,b) and s. Then, with the constraints (16)*

(20) $$\begin{array}{cc} L\left(s\right)-L\left(s^{*}\right)\; & =\frac{1}{2}{∥\Theta {\tilde{B}}_{1}-\Theta W{\left(\Xi_{1}^{X}\right)}^{T}∥}_{F}^{2}+\frac{1}{2}\kappa^{\left(v,b\right)}\left(s,s^{*}\right)+o\left(\epsilon^{2}\right), \end{array}$$

where $\Theta ≜{\left({\left(\Xi^{Y}\right)}^{T}\Xi^{Y}\right)}^{1/2}J$, and the term $\kappa^{\left(v,b\right)}\left(s,s^{*}\right)$$={\left(\mu_{s}-\mu_{s^{*}}\right)}^{T}\Lambda_{\tilde{v}\left(Y\right)}\left(\mu_{s}-\mu_{s^{*}}\right)$.

**Proof.**  See Appendix F. □

Equation (20) quantifies the closeness between *s* and $s^{*}$ in terms of the loss (8). Then, our goal is to minimize (20), which can be separated to two optimization problems:

(21) $$\begin{array}{cc} W^{*} & =\underset{W}{arg min}\;{∥\Theta {\tilde{B}}_{1}-\Theta W{\left(\Xi_{1}^{X}\right)}^{T}∥}_{F}^{2}, \end{array}$$

(22) $$\begin{array}{cc} \mu_{s}^{*} & =\underset{\mu_{s}}{arg min}\;\kappa^{\left(v,b\right)}\left(s,s^{*}\right). \end{array}$$

Note that the optimization problem (21) is similar to the one that appeared in Lemma 3, and the optimal solution is given by $W^{*}={\tilde{B}}_{1}\Xi_{1}^{X}{\left({\left(\Xi_{1}^{X}\right)}^{T}\Xi_{1}^{X}\right)}^{-1}$. Therefore, solving the optimal weights in the hidden layer can be interpreted as projecting ${\tilde{s}}^{*}\left(x\right)$ to the subspace of feature functions spanned by t(x) to find the closest expressible function. In addition, the problem (22) is to choose $\mu_{s}$ (and hence the bias c(z)) to minimize the quadratic term similar to $\eta^{\left(v,b\right)}\left(s\right)$ in (10). Similar to the analyses of parameters in the last layer, we can obtain analytical solutions for hidden layer parameters, e.g., $\mu_{s}^{*}$ and $w^{*}$, with detailed discussions provided in Appendix G.

Overall, we observe the correspondence between (11), (14), and (21), (22), and interpret both operations as feature projections. Our argument can be generalized to any intermediate layer in a multi-layer network, with all the previous layers viewed as the fixed pre-processing that specifies t(x), and all the layers after determining $s^{*}$. Then, the iterative procedure in back-propagation can be viewed as alternating projection finding the fixed-point solution over the entire network. This final fixed-point solution, even under the local assumption, might not be the SVD solution as in Theorem 4. This is because the limited expressive power of the network often makes it impossible to generate the desired feature function. In such cases, the concept of feature projection can be used to quantify this gap, and thus to measure the quality of the selected features.

### 3.3. Scoring Neural Networks

Given a learning problem, it is useful to tell whether or not some extracted features are informative [42]. Our previous development naturally gives rise to a performance metric.

**Definition** **5.** *Given a feature $s\left(x\right)\in R^{k}$ and weight $v\left(y\right)\in R^{k}$ with the corresponding information matrices $\Xi^{X}$ and $\Xi^{Y}$, the H-score H(s,v) is defined as*

(23) $$\begin{array}{c} H(s,v)≜\frac{1}{2}{∥\tilde{B}∥}_{F}^{2}-\frac{1}{2}{∥\tilde{B}-\Xi^{Y}{\left(\Xi^{X}\right)}^{T}∥}_{F}^{2}=E_{P_{XY}}\left[{\tilde{s}}^{T}\left(X\right)\;\tilde{v}\left(Y\right)\right]-\frac{1}{2}\mathrm{tr}\left(\Lambda_{\tilde{s}\left(X\right)}\Lambda_{\tilde{v}\left(Y\right)}\right). \end{array}$$

*In addition, for given s(x), we define the single-sided H-score H(s) as*

(24) $$\begin{array}{c} H(s)≜\max_{v}H\left(s,v\right) \end{array}$$

(25) $$\begin{array}{c} =\frac{1}{2}{∥\tilde{B}∥}_{F}^{2}-\frac{1}{2}{∥\tilde{B}-\tilde{B}\;\Xi^{X}{\left({\left(\Xi^{X}\right)}^{T}\Xi^{X}\right)}^{-1}{\left(\Xi^{X}\right)}^{T}∥}_{F}^{2} \end{array}$$

(26) $$\begin{array}{c} =\frac{1}{2}{∥\tilde{B}\Xi^{X}{\left({\left(\Xi^{X}\right)}^{T}\Xi^{X}\right)}^{-\frac{1}{2}}∥}_{F}^{2}=\frac{1}{2}E_{P_{Y}}\left[{∥E_{P_{X|Y}}\left[\Lambda_{\tilde{s}\left(X\right)}^{-1/2}\;\tilde{s}\left(X\right)|Y\right]∥}^{2}\right]. \end{array}$$

H-score can be used to measure the quality of features generated at any intermediate layer of the network. It is related to (20) when choosing the optimal bias and Θ as the identity matrix. This can be understood as taking the output of this layer s(x) and directly feeding it to a softmax output layer with v(y) used as the weights, and H(s,v) measures the resulting performance. Note that v(y) here can be an arbitrary function of *Y*, not necessarily the weights on the next layer computed by the network. When the optimal $v^{*}\left(y\right)$ as defined in (12) is used, the resulting performance becomes the one-sided H-score H(s), which measures the quality of s(x). In addition, by comparing (26) with (7), the performance measure H(s) also coincides with the information metric (7), up to a scale factor.

Specifically, for a given dataset and a feature extractor that generate s(·), the H-score H(s) can be efficiently computed from the second equation of (26). In addition, when we use H-score to compare the performance of different feature extractors (models), the model complexity has to be taken into account to reduce overfitting. To this end, we adopt Akaike information criterion (AIC) and define *AIC-corrected H-score*

(27) $$\begin{array}{c} H_{\mathrm{AIC}}\left(s\right)≜H\left(s\right)-\frac{n_{p}}{n_{s}} \end{array}$$

for comparing different models, where $n_{p}$ and $n_{s}$ represent the number of parameters in the model and the training sample size, respectively.

In current practice, the cross-entropy $E_{P_{XY}}\left[\log{\tilde{P}}_{Y|X}^{\left(v,b\right)}\right]$ is often used as the performance metric. One can, in principle, also use log-loss to measure the effectiveness of the selected feature at the output of an intermediate layer [42]. However, one problem of this metric is that, for a given problem, it is not clear what value of log-loss one should expect, as the log-loss is generally unbounded. In contrast, the H-score can be directly computed from the data samples and has a clear upper bound. Indeed, it follows from Lemma 1 that, for *k*-dimensional feature *s* and weights *v*, we have the sequence of inequalities

(28) $$\begin{array}{c} H\left(s,v\right)\leq H\left(s\right)\leq \frac{1}{2}\sum_{i=1}^{k}\sigma_{i}^{2}\leq \frac{k}{2}, \end{array}$$

where $\sigma_{i}$ indicates the *i*th singular value of $\tilde{B}$.

In particular, the first “≤” follows from the definition (24), and the gap between H(s,v) and H(v) measures the optimality of the weights *v*; the second “≤” follows from the first equality of (26), and the gap between two sides characterizes the difference between the chosen feature and the optimal solution, which is a useful measure of how restrictive (lack of expressive power) the network structure is; the last “≤” follows from the fact that $\sigma_{i}\leq 1$ (cf. Lemma 1), which measures the dependency between data variable and label for the given dataset. In Section 3.4.3, we validate this metric on real data.

### 3.4. Experiments

This section presents experiments for validating our theoretical characterizations, with corresponding code available at https://github.com/XiangxiangXu/dnn (accessed on 7 December 2021). Specifically, all DNN models used in Section 3.4.3 are available at https://keras.io/applications/ (accessed on 7 December 2021).

#### 3.4.1. Experimental Validation of Theorem 4

**Figure 3:**
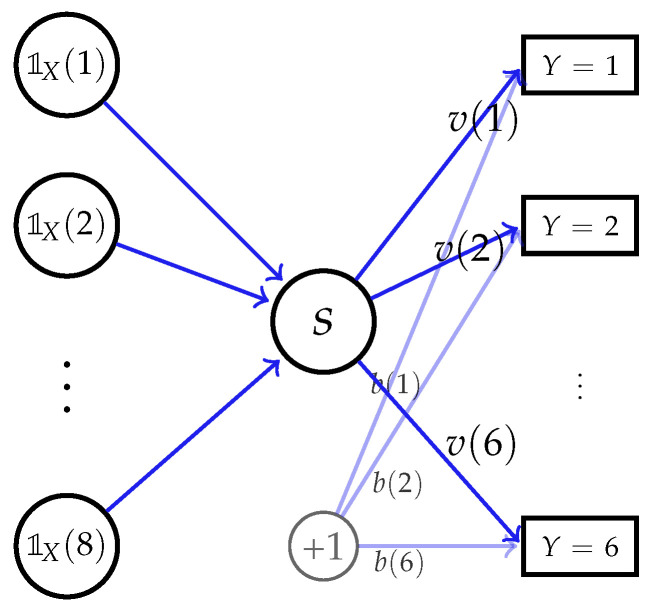
A simple neural network with ideal expressive power, which can generate any k=1 dimensional feature *s* of *X* by tuning the weights in the first layer.

We first validate Theorem 4, the optimal feature extracted by network with ideal expressive power. Here, we consider the discrete data with alphabet sizes, |X|=8 and |Y|=6, and construct the network as shown in Figure 3. Specifically, the network input is the one-hot encoding of *X*, i.e., $[1_{X}\left(1\right),\ldots ,1_{X}{\left(|X|)\right]}^{T}$, where $1_{X}\left(x\right)$ takes one if and only if X=x, and takes zero otherwise. Then, the feature s(X) is generated by a linear layer, with sigmoid function used as the activation function. For ease of comparison and presentation, we set feature dimension to k=1, since otherwise the optimal feature (cf. Theorem 4) lies in a subspace and is non-unique. It can be verified that this network has ideal expressive power, i.e., with proper weights in the first layer, s(X) can express any desired function up to scaling and shifting.

**Figure 4:**
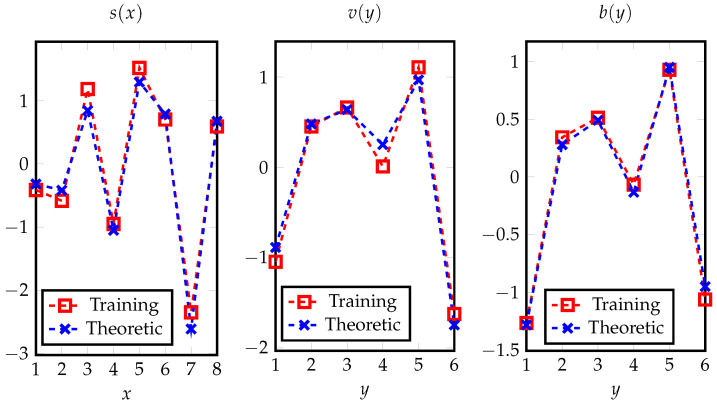
The trained feature *s*, weights *v*, and bias *b* of the network in Figure 3, which are compared with the corresponding theoretical results to show their coincidences.

To compare the result trained by the neural network and that in Theorem 4, we first randomly generate a distribution $P_{XY}$, and then draw independently n= 100,000 pairs of (X,Y) samples. We then train the network using batch gradient descent, where we have applied Nesterov momentum [43] with the momentum hyperparameter being 0.9. In addition, we set the learning rate to 4 with a decay factor of 0.01 and clip gradients with norm exceeding 0.5. After training, the learned values of s(x),v(y) and b(y) are shown in Figure 4 and compared with theoretical results. From the figure, we can observe that the training results match our theoretical analyses.

#### 3.4.2. Experimental Validation of Theorem 5

**Figure 5:**
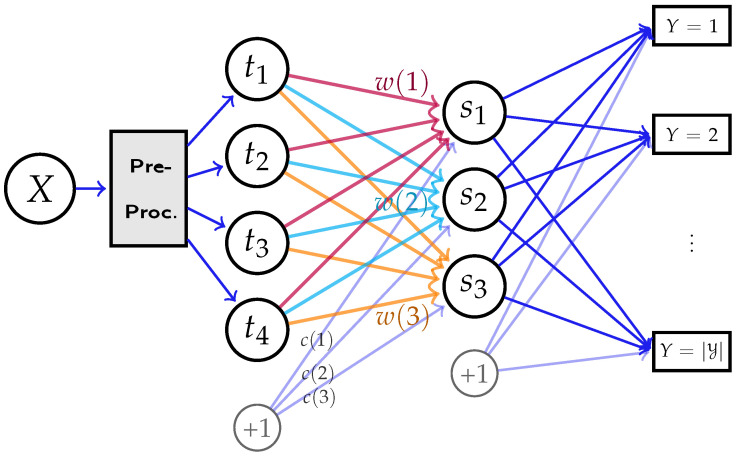
The designed network for validating the impact of network structure on feature extraction, with m=4 and k=3 neurons in two hidden layers. Our goal is to compare the learned weights w(1),w(2), w(3) and bias *c* in the hidden layer with our theoretic characterizations in Section 3.2.2.

**Figure 6:**
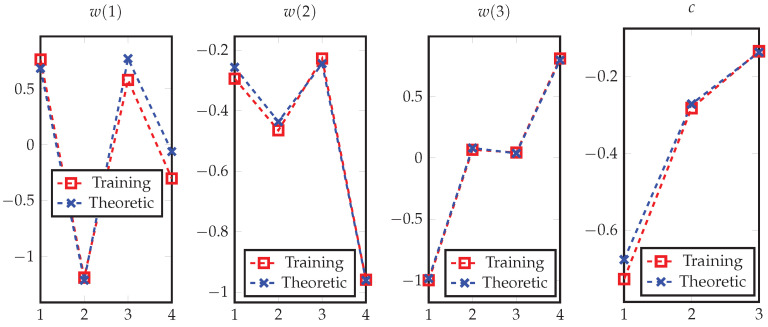
The trained weights *w* and bias *c* of the network in Figure 5, which are compared with the corresponding theoretical results to show their coincidences.

In addition, we validate Theorem 5 by the neural network depicted in Figure 5, with the same settings of X,Y. Specifically, the number of neurons in hidden layers are set to m=4 and k=3, where t(X) is randomly generated from *X*, and we have chosen sigmoid function as the activation function σ(·) to generate s(x). We then fix the weights and bias at the output layer and train the weights w(1),w(2), w(3) and bias *c* in the hidden layer to optimize the log-loss. Specifically, we use the batch gradient descent with the Nesterov momentum hyperparameter being 0.9. In addition, we set the learning rate to 4 with a decay factor of $10^{-6}$ and clip gradients with norm exceeding 0.1. After training, Figure 6 shows the matching between the learned results and the corresponding theoretical values.

#### 3.4.3. Experimental Validation of H-Score

To validate H-score as a performance measure for extracted features, we compare the H-score and classification accuracy of DNNs on image classification tasks. Specifically, we use the ImageNet Large Scale Visual Recognition Challenge 2012 (ILSVRC2012) [22] dataset as the dataset and extract features using several deep neural networks with representative architectures designs [44,45,46,47,48,49]. After training the feature extractors on the ILSVRC2012 training set, we then compute the H-score of the feature in the last hidden layer, as well as the classification accuracies on ILSVRC2012 validation set (here, we use ILSVRC2012 validation set for testing, as the labels in ILSVRC2012 testing set have not been publicly released). The results are summarized in Table 1, where $H_{\mathrm{AIC}}\left(s\right)$ is the AIC-corrected H-score as defined in (27), with $n_{p}$ being the number of model parameters, and $n_{s}=$ 1,300,000 corresponding to the number of training samples in ImageNet. The AIC-corrected H-score is consistent with the classification accuracy, which validates the effectiveness of H-score as a measurement of neural networks.

## 4. Discussion

Our characterization gives an information-theoretic interpretation of the feature extraction process in DNNs, which also provides a practical performance measure for scoring neural networks. Different from empirical studies focusing on specific datasets [7], our development is based on the probability distribution space, which is more general and can also provide theoretic insights. Moreover, the information-theoretic framework allows us to obtain direct operational meaning and better interpretations for the solutions, compared with optimization-based theoretical characterizations, e.g., [11,13].

As a first step in establishing a rigorous framework for DNN analysis, the present work can be extended in both theoretical and practical aspects. From the theoretical perspective, one extension is to investigate the analytical properties for general DNNs, using the theoretic insights obtained from local analysis regime. For example, it was shown in [50] that the symmetry between feature and weights in DNNs established in the local analysis regime (cf. Section 3.2.1) also holds for general probability distributions. Another extension is to apply the framework to investigate the optimal feature for structured data or network, e.g., data with sparsity structure [51].

From the practical perspective, in addition to the demonstrated example of evaluating existing DNN models (cf. Section 3.4.3), the H-score can also be used as an objective function in designing learning algorithms. In particular, such usages have been illustrated in multi-modal learning [52] and transfer learning [53] tasks.

## 5. Conclusions

In this paper, we apply the local information geometric analysis and provide an information-theoretic interpretation to the feature extraction scheme in DNNs. We first establish an information metric for features in inference tasks by formalizing the information-theoretic feature selection problem. In addition, we demonstrate that the features extracted by DNNs coincide with the information-theoretically optimal feature, with the same metric measuring the performance of features, called H-score. Furthermore, we discuss the usage of the H-score for measuring the effectiveness of DNNs. Our framework demonstrates a connection between the practical deep learning implementations and information-theoretic characterizations, which can provide theoretical insights for DNN analysis and learning algorithm designs.

## Figures and Tables

**Table 1:** Classification accuracy and H-score for different DNN models on ImageNet dataset, where “Paras” indicates the number of parameters (in millions) in the model and $H_{\mathrm{AIC}}$ represents the AIC-corrected H-score.

DNN Model	Paras [$\times 10^{6}$]	H(s)	$H_{\mathrm{AIC}}\left(s\right)$	Accuracy [%]
VGG16 [44]	138.4	148.3	41.9	64.2
VGG19 [44]	143.7	152.7	42.2	64.7
MobileNet [45]	4.3	45.9	42.6	68.4
DenseNet121 [46]	8.1	59.5	53.3	71.4
DenseNet169 [46]	14.3	81.2	70.2	73.6
DenseNet201 [46]	20.2	89.1	73.5	74.4
Xception [47]	22.9	179.8	162.2	77.5
InceptionV3 [48]	23.9	181.2	162.9	76.3
InceptionResNetV2 [49]	55.9	241.1	198.1	79.1

## Appendix A. Proof of Theorem 1

We commence with the characterization of the error exponent.

**Lemma** **A1.**
*Given a reference distribution $P_{X}\in \mathrm{relint}\left(P^{X}\right)$, a constant ϵ>0 and integers n and k, let $x_{1},\ldots ,x_{n}$ denote i.i.d. samples from one of $P_{1}$ or $P_{2}$, where $P_{1},P_{2}\in N_{\epsilon }^{X}\left(P_{X}\right)$. To decide whether $P_{1}$ or $P_{2}$ is the generating distribution, a sequence of k-dimensional statistics $h^{k}=\left(h_{1},\ldots ,h_{k}\right)$ is constructed as*

(A1)
$$h_{i}=\frac{1}{n}\sum_{l=1}^{n}f_{i}\left(x_{l}\right),\;i=1,\ldots ,k,$$

*where $\left(f_{1}\left(X\right),\ldots ,f_{k}\left(X\right)\right)$ are zero mean, unit-variance, and uncorrelated with respect to $P_{X}$, i.e.,*

(A2)
$$\begin{array}{c} E_{P_{X}}\left[f_{i}\left(X\right)\right]=0,\;i\in \left\{1,\ldots ,k\right\} \end{array}$$

(A3)
$$\begin{array}{c} E_{P_{X}}\left[f_{i}\left(X\right)f_{j}\left(X\right)\right]=\delta_{ij},\;i,j\in \left\{1,\ldots ,k\right\}. \end{array}$$

*Then, the error probability of the decision based on $h^{k}$ decays exponentially in n as n→∞, with (Chernoff) exponent*

(A4)
$$\lim_{n\to \infty }\frac{-\log p_{e}}{n}≜E_{h^{k}}=\sum_{i=1}^{k}E_{h_{i}},$$

*where*

(A5)
$$E_{h_{i}}=\frac{1}{8}{\langle \phi_{1}-\phi_{2},\xi_{i}\rangle }^{2}+o\left(\epsilon^{2}\right),$$

*and $\phi_{1}↔P_{1},\phi_{2}↔P_{2},\xi_{i}↔f_{i}\left(X\right),i\in \left\{1,\ldots ,k\right\}$ are the corresponding information vectors.*

**Proof** **of** **Lemma** **A1.** Since the rule is to decide based on comparing the projection

$$\sum_{i=1}^{k}h_{i}\left(E_{P_{1}}\left[f_{i}\left(X\right)\right]-E_{P_{2}}\left[f_{i}\left(X\right)\right]\right)$$

to a threshold, via Cramér’s theorem [54], the error exponent under $P_{j}\;\left(j=1,2\right)$ is

(A6) $$E_{j}\left(\lambda \right)=\min_{P\in S(\lambda )}D\left(P∥P_{j}\right),$$

where

(A7) $$\begin{array}{cc} \; & S\left(\lambda \right)≜\left\{P\in P^{X}:E_{P}\left[f^{k}\left(X\right)\right]=\lambda \;E_{P_{1}}\left[f^{k}\left(X\right)\right]+\left(1-\lambda \right)\;E_{P_{2}}\left[f^{k}\left(X\right)\right]\right\}. \end{array}$$

Now, since (A2) holds, we obtain

(A8) $$\begin{array}{cc} E_{P_{j}}\left[f_{i}\left(X\right)\right]\; & =\sum_{x\in X}P_{j}\left(x\right)\;f_{i}\left(x\right)\\ \; & =\sum_{x\in X}P_{X}\left(x\right)\;f_{i}\left(x\right)+\sum_{x\in X}\left(P_{j}\left(x\right)-P_{X}\left(x\right)\right)f_{i}\left(x\right)\\ \; & =E_{P_{X}}\left[f_{i}\left(X\right)\right]+\sum_{x\in X}\sqrt{P_{X}\left(x\right)}\;\phi_{j}\left(x\right)\cdot \frac{\xi_{i}\left(x\right)}{\sqrt{P_{X}\left(x\right)}}\\ \; & =\sum_{x\in X}\phi_{j}\left(x\right)\;\xi_{i}\left(x\right)\\ \; & =\langle \phi_{j},\xi_{i}\rangle ,\;j=1,2\;\mathrm{and}\;i=1,\ldots ,k, \end{array}$$

which we express compactly as

$$E_{P_{j}}\left[f^{k}\left(X\right)\right]=\langle \phi_{j},\xi^{k}\rangle ,\;j=1,2$$

with $\xi^{k}≜\left(\xi_{1},\ldots ,\xi_{k}\right)$. Hence, the constraint (A7) is expressed in information vectors as

$$\langle \phi ,\xi_{i}\rangle =\langle \lambda \;\phi_{1}+\left(1-\lambda \right)\;\phi_{2},\xi_{i}\rangle ,\;i=1,\cdots ,k,$$

i.e.,

(A9) $$\langle \phi ,\xi^{k}\rangle =\langle \lambda \;\phi_{1}+\left(1-\lambda \right)\;\phi_{2},\xi^{k}\rangle .$$

In turn, the optimal *P* in (A6), which we denoted by $P^{*}$, lies in the exponential family through $P_{j}$ with natural statistic $f^{k}\left(x\right)$, i.e., the *k*-dimensional family whose members are of the form

$$\log{\tilde{P}}_{\theta^{k}}\left(x\right)=\sum_{i=1}^{k}\theta_{i}f_{i}\left(x\right)+\log P_{j}\left(x\right)-\alpha \left(\theta^{k}\right),$$

for which the associated information vector is

(A10) $${\tilde{\phi }}_{\theta^{k}}\left(x\right)=\sum_{i=1}^{k}\theta_{i}\xi_{i}\left(x\right)+\phi_{j}\left(x\right)-\alpha \left(\theta^{k}\right)\sqrt{P_{X}\left(x\right)}+o\left(\epsilon \right),$$

where we have used the fact that

$$\begin{array}{cc} \log Q_{X}\left(x\right) & =\log P_{X}\left(x\right)+\log\frac{Q_{X}\left(x\right)}{P_{X}\left(x\right)}\\ \; & =\log P_{X}\left(x\right)+\log\left(1+\frac{1}{\sqrt{P_{X}\left(x\right)}}\phi \left(x\right)\right)\\ \; & =\log P_{X}\left(x\right)+\frac{1}{\sqrt{P_{X}\left(x\right)}}\phi \left(x\right)+o\left(\epsilon \right) \end{array}$$

for all $Q_{X}\in N_{\epsilon }^{X}\left(P_{X}\right)$ with the information vector $\phi ↔Q_{X}$. As a result,

$$\langle {\tilde{\phi }}_{\theta^{k}},\xi_{i}\rangle =\theta_{i}+\langle \phi_{j},\xi_{i}\rangle +o\left(\epsilon \right),$$

where we have used (A3). Hence, via (A9), we obtain that the intersection with the linear family (A7) is at $P^{*}=P_{{\theta^{k}}^{*}}$ with

$$\theta_{i}^{*}=\langle \lambda \phi_{1}+\left(1-\lambda \right)\phi_{2}-\phi_{j},\xi_{i}\rangle +o\left(\epsilon \right)$$

and thus

$$\begin{array}{cc} E_{j}\left(\lambda \right)\; & =D(P^{*}∥P_{j}) \end{array}$$

(A11) $$\begin{array}{cc} \; & =\frac{1}{2}{∥{\tilde{\phi }}_{\theta^{k}}-\phi_{j}∥}^{2}+o\left(\epsilon^{2}\right) \end{array}$$

(A12) $$\begin{array}{cc} \; & =\frac{1}{2}{∥\sum_{i=1}^{k}\theta_{i}^{*}\xi_{i}∥}^{2}+\frac{1}{2}\alpha {\left({\theta^{k}}^{*}\right)}^{2}+o\left(\epsilon^{2}\right) \end{array}$$

(A13) $$\begin{array}{cc} \; & =\frac{1}{2}\sum_{i=1}^{k}{\left(\theta_{i}^{*}\right)}^{2}+\frac{1}{2}\alpha {\left({\theta^{k}}^{*}\right)}^{2}+o\left(\epsilon^{2}\right) \end{array}$$

(A14) $$\begin{array}{cc} \; & =\frac{1}{2}\sum_{i=1}^{k}{\langle \lambda \phi_{1}+\left(1-\lambda \right)\phi_{2}-\phi_{j},\xi_{i}\rangle }^{2}+o\left(\epsilon^{2}\right), \end{array}$$

where to obtain (A11) we have exploited the local approximation of KL divergence [18], to obtain (A12) we have exploited (A10), to obtain (A13) we have again exploited (A3), and to obtain (A14) we have used that

$$\alpha \left({\theta^{k}}^{*}\right)=o\left(\epsilon^{2}\right)$$

since ${\theta^{k}}^{*}=O\left(\epsilon \right)$ and

$$\alpha \left(0\right)=0,\;\mathrm{and}\;\nabla \alpha \left(0\right)=E_{P_{j}}\left[f^{k}\left(X\right)\right]=\langle \phi_{j},\xi^{k}\rangle =O\left(\epsilon \right).$$

Finally, $E_{1}\left(\lambda \right)=E_{2}\left(\lambda \right)$ when λ=1/2, so the overall error probability has exponent (A5). □

Then, the following lemma demonstrates a property of information vectors in a Markov chain.

**Lemma** **A2.**
*Given the Markov relation X↔Y↔V and any v∈V, let $\phi_{v}^{X|V}$ and $\phi_{v}^{Y|V}$ denote the associated information vectors for $P_{X|V}\left(\cdot |v\right)$ and $P_{Y|V}\left(\cdot |v\right)$, then we have*

(A15)
$$\phi_{v}^{X|V}={\tilde{B}}^{T}\phi_{v}^{Y|V}.$$

**Proof** **of** **Lemma** **A2.** From the Markov relation we have

$$P_{X}\left(x\right)=\sum_{y\in Y}P_{X|Y}\left(x|y\right)P_{Y}\left(y\right)$$

and

$$\begin{array}{c} P_{X|V}\left(x|v\right)=\sum_{y\in Y}P_{X|Y,V}\left(x|y,v\right)P_{Y|V}\left(y|v\right)=\sum_{y\in Y}P_{X|Y}\left(x|y\right)P_{Y|V}\left(y|v\right). \end{array}$$

As a result,

$$P_{X|V}\left(x|v\right)-P_{X}\left(x\right)=\sum_{y\in Y}P_{X|Y}\left(x|y\right)\left[P_{Y|V}\left(y|v\right)-P_{Y}\left(y\right)\right],$$

from which we obtain the corresponding information vector

(A16) $$\begin{array}{cc} \phi_{v}^{X|V}\left(x\right)\; & =\frac{1}{\sqrt{P_{X}\left(x\right)}}\sum_{y\in Y}P_{X|Y}\left(x|y\right)\sqrt{P_{Y}\left(y\right)}\phi_{v}^{Y|V}\left(y\right)\\ \; & =\sum_{y\in Y}\left[\tilde{B}\left(y,x\right)+\sqrt{P_{X}\left(x\right)P_{Y}\left(y\right)}\right]\phi_{v}^{Y|V}\left(y\right)\\ \; & =\sum_{y\in Y}\tilde{B}\left(y,x\right)\phi_{v}^{Y|V}\left(y\right), \end{array}$$

where the last equality follows from the fact that

$$\sum_{y\in Y}\sqrt{P_{Y}\left(y\right)}\phi_{v}^{Y|V}\left(y\right)=\sum_{y\in Y}\left[P_{Y|V}\left(y|v\right)-P_{Y}\left(y\right)\right]=0.$$

Finally, rewrite (A16) in the matrix form and we obtain (A15). □

In addition, the following lemma is useful for dealing with the expectation over an RIE.

**Lemma** **A3.**
*Let z be a spherically symmetric random vector of dimension M, i.e., for any orthogonal Q we have $z\overset{d}{=}Qz$. If A is a fixed matrix of compatible dimensions, then*

(A17)
$$E\left[∥z^{T}{A∥}^{2}\right]=\frac{1}{M}E\left[{∥z∥}^{2}\right]{∥A∥}_{F}^{2}.$$

**Proof** **of** **Lemma** **A3.** By definition we have $\Lambda_{z}=Q\Lambda_{z}Q^{T}$ for any orthogonal Q; hence, $\Lambda_{z}$ is diagonal. Suppose $\Lambda_{z}=\lambda \;I$, then from

$$\mathrm{tr}\left(\Lambda_{z}\right)=E\left[{∥z∥}^{2}\right]=\lambda M$$

we obtain

$$\lambda =\frac{1}{M}\mathrm{tr}\left(\Lambda_{z}\right).$$

As a result, we have

$$\begin{array}{cc} E\left[∥z^{T}{A∥}^{2}\right] & =\mathrm{tr}\left(A^{T}\Lambda_{z}A\right)=\lambda \mathrm{tr}\left(A^{T}A\right)=\frac{1}{M}E\left[{∥z∥}^{2}\right]{∥A∥}_{F}^{2}. \end{array}$$

□

Proceeding to our proof of Theorem 1, by definition of feature functions, we have $E_{P_{X}}\left[f_{i}\left(X\right)\right]=0,i=1,\ldots ,k$. Suppose f is the vector representation of $f^{k}$ and denote by $\tilde{f}≜\Lambda_{f}^{-1/2}f$ the normalized f, with $\Lambda_{f}^{1/2}$ denoting any square root matrix of $\Lambda_{f}$. Then, the corresponding statistics ${\tilde{f}}^{k}=\left({\tilde{f}}_{1},\ldots ,{\tilde{f}}_{k}\right)$ satisfy the constraints (A2) and (A3). In addition, we construct the statistic ${\tilde{h}}^{k}=\left({\tilde{h}}_{1},\ldots ,{\tilde{h}}_{k}\right)$ as [cf. (A1)]

(A18) $${\tilde{h}}_{i}=\frac{1}{n}\sum_{l=1}^{n}{\tilde{f}}_{i}\left(x_{l}\right),\;i=1,\ldots ,k.$$

Then, from Lemma A1, the error exponent of distinguishing *v* and $v^{\prime }$ based on ${\tilde{h}}^{k}$ is

$$\begin{array}{cc} E_{{\tilde{h}}^{k}}\left(v,v^{\prime }\right)\; & =\frac{1}{8}\sum_{i=1}^{k}{\left[{\left(\phi_{v}^{X|V}-\phi_{v^{\prime }}^{X|V}\right)}^{T}{\tilde{\xi }}_{i}^{X}\right]}^{2}+o\left(\epsilon^{2}\right)\\ \; & =\frac{1}{8}{∥{\left(\phi_{v}^{X|V}-\phi_{v^{\prime }}^{X|V}\right)}^{T}{\tilde{\Xi }}^{X}∥}^{2}+o\left(\epsilon^{2}\right), \end{array}$$

where $\phi_{v}^{X|V}$ denotes the associated information vector for $P_{X|V}\left(\cdot |v\right)$, ${\tilde{\xi }}_{i}^{X}$ denotes the information vectors of ${\tilde{f}}_{i}$, and ${\tilde{\Xi }}^{X}≜\left[{\tilde{\xi }}_{1}^{X},\ldots ,{\tilde{\xi }}_{k}^{X}\right]$. Since the optimal decision rule is linear, the error exponent is invariant with linear transformations of statistics, i.e.,

(A19) $$\begin{array}{cc} E_{h^{k}}\left(v,v^{\prime }\right)=E_{{\tilde{h}}^{k}}\left(v,v^{\prime }\right)\; & =\frac{1}{8}{∥{\left(\phi_{v}^{X|V}-\phi_{v^{\prime }}^{X|V}\right)}^{T}{\tilde{\Xi }}^{X}∥}^{2}+o\left(\epsilon^{2}\right)\\ \; & =\frac{1}{8}{∥{\left(\phi_{v}^{Y|V}-\phi_{v^{\prime }}^{Y|V}\right)}^{T}\tilde{B}{\tilde{\Xi }}^{X}∥}^{2}+o\left(\epsilon^{2}\right), \end{array}$$

where the last equality follows from Lemma A2.

As a result, taking the expectation of (A19) over a given RIE yields

$$\begin{array}{cc} E\left[E_{h^{k}}\left(v,v^{\prime }\right)\right]\; & =\frac{1}{8}E\left[{∥{\left(\phi_{v}^{Y|V}-\phi_{v^{\prime }}^{Y|V}\right)}^{T}\tilde{B}{\tilde{\Xi }}^{X}∥}^{2}\right]+o\left(\epsilon^{2}\right)\\ \; & =\frac{E\left[∥\phi_{v}^{Y|V}-\phi_{v^{\prime }}^{Y|V}{∥}^{2}\right]}{8|Y|}{∥\tilde{B}{\tilde{\Xi }}^{X}∥}_{F}^{2}+o\left(\epsilon^{2}\right), \end{array}$$

where we have exploited Lemma A3. Finally, the error exponent (7) can be obtained via noting from the definition of ${\tilde{f}}^{k}$ that

$${\tilde{\Xi }}^{X}=\Xi^{X}{\left({\left(\Xi^{X}\right)}^{T}\Xi^{X}\right)}^{-\frac{1}{2}}.$$

## Appendix B. Proof of Lemma 2

We first prove two useful lemmas.

**Lemma** **A4.**
*For distributions $P\in \mathrm{relint}(P^{X})$, $Q,R\in P^{X}$, and sufficiently small ϵ, if $D\left(P∥Q\right)\leq \epsilon^{2}$ and $D\left(P∥R\right)\leq \epsilon^{2}$, then there exists a constant C>0 independent of ϵ, such that $D\left(Q∥R\right)\leq C\epsilon^{2}$.*

**Proof** **of** **Lemma** **A4.** Denote by ${∥\cdot ∥}_{1}$ the $\ell_{1}$-distance between distributions, i.e., ${∥P-Q∥}_{1}≜\sum_{x\in X}\left|P\left(x\right)-Q\left(x\right)\right|$, then from Pinsker’s inequality [14], we have

(A20) $$\begin{array}{cc} {∥P-Q∥}_{1} & \leq \sqrt{2D(P∥Q)}<\sqrt{2}\epsilon , \end{array}$$

(A21) $$\begin{array}{cc} {∥P-R∥}_{1} & \leq \sqrt{2D(P∥R)}<\sqrt{2}\epsilon , \end{array}$$

which implies

(A22) $$\begin{array}{c} {∥Q-R∥}_{1}\leq {∥P-Q∥}_{1}+{∥P-R∥}_{1}\leq 2\sqrt{2}\epsilon . \end{array}$$

In addition, with $p_{\min}≜\min_{x\in X}P\left(x\right)$, for all x∈X we have

(A23) $$\begin{array}{cc} R(x) & >P(x)-|P(x)-R(x)| \end{array}$$

(A24) $$\begin{array}{cc} \; & >\min_{x\in X}P\left(x\right)-\sqrt{2}\epsilon \end{array}$$

(A25) $$\begin{array}{cc} \; & =p_{\min}-\sqrt{2}\epsilon , \end{array}$$

where to obtain (A24) we have used (A21). Note that since $P\in \mathrm{relint}(P^{X})$ we have $p_{\min}>0$, and thus $R\left(x\right)>p_{\min}/2$ for sufficiently small ϵ. As a result,

(A26) $$\begin{array}{cc} D(Q∥R)\; & \leq \sum_{x\in X}\frac{{\left(Q\left(x\right)-R\left(x\right)\right)}^{2}}{R(x)} \end{array}$$

(A27) $$\begin{array}{cc} \; & \leq \frac{2}{p_{\min}}\sum_{x\in X}{\left[Q\left(x\right)-R\left(x\right)\right]}^{2} \end{array}$$

(A28) $$\begin{array}{cc} \; & \leq \frac{{2∥Q-R∥}_{1}^{2}}{p_{\min}} \end{array}$$

(A29) $$\begin{array}{cc} \; & \leq \frac{16}{p_{\min}}\epsilon^{2}, \end{array}$$

where to obtain (A26) we have used the fact that KL divergence is upper bounded by corresponding $\chi^{2}$-divergence [55], and to obtain (A29) we have used (A22). □

**Lemma** **A5.**
*For all (x,y)∈X×Y, we have*

$$\begin{array}{c} D\left(P_{X}P_{Y}∥P_{X}\;{\tilde{P}}_{Y|X}^{\left(s,v,b\right)}\right)\geq P_{X}\left(x\right)\log\left[P_{Y}\left(y\right)e^{\tau (x,y)}+\left(1-P_{Y}\left(y\right)\right)e^{-\frac{P_{Y}\left(y\right)}{1-P_{Y}\left(y\right)}\tau \left(x,y\right)}\right] \end{array}$$

*where ${\tilde{P}}_{Y|X}^{\left(s,v,b\right)}$ is as defined in (4), and where we have defined $\tau \left(x,y\right)≜{\tilde{v}}^{T}\left(y\right)s\left(x\right)+\tilde{d}\left(y\right)$.*

**Proof** **of** **Lemma** **A5.** First, we can rewrite the conditional distribution ${\tilde{P}}_{Y|X}^{\left(s,v,b\right)}\left(y|x\right)$ as

(A30) $$\begin{array}{cc} {\tilde{P}}_{Y|X}^{\left(s,v,b\right)}\left(y|x\right)=\frac{e^{v^{T}\left(y\right)s\left(x\right)+b\left(y\right)}}{\sum_{y^{\prime }\in Y}e^{v^{T}\left(y^{\prime }\right)s\left(x\right)+b\left(y^{\prime }\right)}}\; & =\frac{P_{Y}\left(y\right)e^{v^{T}\left(y\right)s\left(x\right)+d\left(y\right)}}{\sum_{y^{\prime }\in Y}P_{Y}\left(y^{\prime }\right)e^{v^{T}\left(y^{\prime }\right)s\left(x\right)+d\left(y^{\prime }\right)}}\\ \; & =\frac{P_{Y}\left(y\right)e^{{\tilde{v}}^{T}\left(y\right)s\left(x\right)+\tilde{d}\left(y\right)}}{\sum_{y^{\prime }\in Y}P_{Y}\left(y^{\prime }\right)e^{{\tilde{v}}^{T}\left(y^{\prime }\right)s\left(x\right)+\tilde{d}\left(y^{\prime }\right)}}\\ \; & =\frac{P_{Y}\left(y\right)e^{\tau (x,y)}}{\sum_{y^{\prime }\in Y}P_{Y}\left(y^{\prime }\right)e^{\tau (x,y^{\prime })}}. \end{array}$$

Then, the KL divergence $D(P_{X}P_{Y}∥P_{X}\;{\tilde{P}}_{Y|X}^{\left(s,v,b\right)})$ can be expressed as

(A31) $$\begin{array}{cc} D(P_{X}P_{Y}∥P_{X}\;{\tilde{P}}_{Y|X}^{\left(s,v,b\right)})\; & =\sum_{(x,y)\in X\times Y}P_{X}\left(x\right)P_{Y}\left(y\right)\log\frac{\sum_{y^{\prime }\in Y}P_{Y}\left(y^{\prime }\right)e^{\tau (x,y^{\prime })}}{e^{\tau (x,y)}}\\ \; & =\sum_{x\in X}P_{X}\left(x\right)\log\left[\sum_{y^{\prime }\in Y}P_{Y}\left(y^{\prime }\right)e^{\tau (x,y^{\prime })}\right]-E_{P_{X}P_{Y}}\left[\tau (X,Y)\right]\\ \; & =\sum_{x\in X}P_{X}\left(x\right)\log\left[\sum_{y^{\prime }\in Y}P_{Y}\left(y^{\prime }\right)e^{\tau (x,y^{\prime })}\right], \end{array}$$

where to obtain the last equality we have used the fact $E_{P_{X}P_{Y}}\left[\tau (X,Y)\right]=0$. As a result, we have

(A32) $$\begin{array}{cc} D(P_{X}P_{Y}∥P_{X}\;{\tilde{P}}_{Y|X}^{\left(s,v,b\right)}) & \geq P_{X}\left(x\right)\log\left[\sum_{y^{\prime }\in Y}P_{Y}\left(y^{\prime }\right)e^{\tau (x,y^{\prime })}\right] \end{array}$$

(A33) $$\begin{array}{cc} \; & \geq P_{X}\left(x\right)\log\left[P_{Y}\left(y\right)e^{\tau (x,y)}+\left(1-P_{Y}\left(y\right)\right)e^{-\frac{P_{Y}\left(y\right)}{1-P_{Y}\left(y\right)}\tau \left(x,y\right)}\right], \end{array}$$

where the last inequality follows from Jensen’s inequality:

$$\begin{array}{cc} \sum_{y^{\prime }\in Y}P_{Y}\left(y^{\prime }\right)e^{\tau (x,y^{\prime })}\; & =P_{Y}\left(y\right)e^{\tau (x,y)}+\left(1-P_{Y}\left(y\right)\right)\sum_{y^{\prime }\neq y}\frac{P_{Y}\left(y^{\prime }\right)}{1-P_{Y}\left(y\right)}e^{\tau (x,y^{\prime })}\\ \; & \geq P_{Y}\left(y\right)e^{\tau (x,y)}+\left(1-P_{Y}\left(y\right)\right)\exp\left(\frac{1}{1-P_{Y}\left(y\right)}\sum_{y^{\prime }\neq y}P_{Y}\left(y^{\prime }\right)\tau \left(x,y^{\prime }\right)\right)\\ \; & =P_{Y}\left(y\right)e^{\tau (x,y)}+\left(1-P_{Y}\left(y\right)\right)e^{-\frac{P_{Y}\left(y\right)}{1-P_{Y}\left(y\right)}\tau \left(x,y\right)}. \end{array}$$

□

Proceeding to our proof of Lemma 2, first note that when v=d=0, we have ${\tilde{P}}_{Y|X}^{\left(s,v,b\right)}=P_{Y}$. As a result, the optimal v,d for (8) satisfy

(A34) $$\begin{array}{cc} D(P_{XY}∥P_{X}\;{\tilde{P}}_{Y|X}^{\left(s,v,b\right)})\; & \leq D(P_{XY}∥P_{X}P_{Y})\\ \; & \leq \sum_{(x,y)\in X\times Y}\frac{{\left[P_{X,Y}\left(x,y\right)-P_{X}\left(x\right)P_{Y}\left(y\right)\right]}^{2}}{P_{X}\left(x\right)P_{Y}\left(y\right)}\\ \; & \leq \epsilon^{2}, \end{array}$$

where to obtain the second inequality we have again exploited $\chi^{2}$-divergence as an upper bound of KL divergence [55], and to obtain the last inequality we have used the definition of ϵ-dependency.

As $P_{XY}\in \mathrm{relint}\left(P^{X\times Y}\right)$, from Lemma A4, there exist C>0 and $\epsilon_{1}>0$ such that $D\left(P_{X}P_{Y}∥P_{X}\;{\tilde{P}}_{Y|X}^{\left(s,v,b\right)}\right)<C\epsilon^{2}$ for all $\epsilon <\epsilon_{1}$. Furthermore, from Lemma A5, for all (x,y)∈X×Y and $\epsilon \in (0,\epsilon_{1})$, we have

(A35) $$\begin{array}{cc} C\epsilon^{2}\; & \geq P_{X}\left(x\right)\log\left[P_{Y}\left(y\right)e^{\tau (x,y)}+\left(1-P_{Y}\left(y\right)\right)e^{-\frac{P_{Y}\left(y\right)}{1-P_{Y}\left(y\right)}\tau \left(x,y\right)}\right]. \end{array}$$

Note that the right-hand side of (A35) satisfies

$$\begin{array}{c} \log\left[P_{Y}\left(y\right)e^{\tau (x,y)}+\left(1-P_{Y}\left(y\right)\right)e^{-\frac{P_{Y}\left(y\right)}{1-P_{Y}\left(y\right)}\tau \left(x,y\right)}\right]=\frac{P_{Y}\left(y\right)}{2(1-P_{Y}\left(y\right))}\tau^{2}\left(x,y\right)+o\left(\tau^{2}\left(x,y\right)\right). \end{array}$$

Therefore, there exists δ>0 independent of $\epsilon_{1}$, such that for all |τ(x,y)|≤δ, we have

(A36) $$\begin{array}{c} \log\left[P_{Y}\left(y\right)e^{\tau (x,y)}+\left(1-P_{Y}\left(y\right)\right)e^{-\frac{P_{Y}\left(y\right)}{1-P_{Y}\left(y\right)}\tau \left(x,y\right)}\right]>\frac{P_{Y}\left(y\right)}{2}\tau^{2}\left(x,y\right). \end{array}$$

In addition, if |τ(x,y)|>δ, we have

$$\begin{array}{cc} \; & \log\left[P_{Y}\left(y\right)e^{\tau (x,y)}+\left(1-P_{Y}\left(y\right)\right)e^{-\frac{P_{Y}\left(y\right)}{1-P_{Y}\left(y\right)}\tau \left(x,y\right)}\right]\\ \; & \geq \min\left\{\log\left[P_{Y}\left(y\right)e^{\delta }+\left(1-P_{Y}\left(y\right)\right)e^{-\frac{P_{Y}\left(y\right)}{1-P_{Y}\left(y\right)}\delta }\right],\log\left[P_{Y}\left(y\right)e^{-\delta }+\left(1-P_{Y}\left(y\right)\right)e^{\frac{P_{Y}\left(y\right)}{1-P_{Y}\left(y\right)}\delta }\right]\right\}\\ \; & \geq \frac{P_{Y}\left(y\right)}{2}\delta^{2}, \end{array}$$

where to obtain the second inequality we have exploited the monotonicity of function $t\mapsto P_{Y}\left(y\right)e^{t}+\left(1-P_{Y}\left(y\right)\right)e^{-\frac{P_{Y}\left(y\right)}{1-P_{Y}\left(y\right)}t}$, and to obtain the third inequality we have exploited (A36).

As a result, we have

(A37) $$\begin{array}{c} \log\left[P_{Y}\left(y\right)e^{\tau (x,y)}+\left(1-P_{Y}\left(y\right)\right)e^{-\frac{P_{Y}\left(y\right)}{1-P_{Y}\left(y\right)}\tau \left(x,y\right)}\right]>\frac{P_{Y}\left(y\right)}{2}\cdot \min\left\{\delta^{2},\tau^{2}\left(x,y\right)\right\}. \end{array}$$

Hence, (A35) becomes

(A38) $$\begin{array}{c} C\epsilon^{2}\geq \frac{P_{X}\left(x\right)P_{Y}\left(y\right)}{2}\cdot \min\left\{\delta^{2},\tau^{2}\left(x,y\right)\right\}, \end{array}$$

from which we can obtain τ(x,y)=O(ϵ). To see this, let

$$\begin{array}{c} \epsilon_{2}≜\frac{\delta }{\sqrt{2C}}\cdot \min_{(x,y)\in X\times Y}\sqrt{P_{X}\left(x\right)P_{Y}\left(y\right)},\;\epsilon_{0}≜\min\left\{\epsilon_{1},\epsilon_{2}\right\}. \end{array}$$

Then, for all $\epsilon <\epsilon_{0}$, we have

$$C\epsilon^{2}<\frac{P_{X}\left(x\right)P_{Y}\left(y\right)}{2}\cdot \delta^{2},$$

and (A38) implies $|\tau \left(x,y\right)|<C^{\prime }\epsilon$ with $C^{\prime }=\sqrt{\frac{2C}{P_{X}\left(x\right)P_{Y}\left(y\right)}}$.

## Appendix C. Proof of Lemma 3

**Proof.** From Lemma 2, there exists $C^{\prime }>0$ such that for all (x,y)∈X×Y, we have

(A39) $$|{\tilde{v}}^{T}\left(y\right)s\left(x\right)+\tilde{d}\left(y\right)|<C^{\prime }\epsilon ,$$

which implies

(A40) $$\begin{array}{c} |\mu_{s}^{T}\tilde{v}\left(y\right)+\tilde{d}\left(y\right)|<C\epsilon , \end{array}$$

(A41) $$\begin{array}{c} |{\tilde{v}}^{T}\left(y\right)\tilde{s}\left(x\right)|<2C\epsilon , \end{array}$$

with $C=\max\{C^{\prime },1\}$. From (A30), we can assume $E_{P_{Y}}\left[v(Y)\right]=E_{P_{Y}}\left[d(Y)\right]=0$ without loss of generality. Then, (4) can be rewritten as

(A42) $$\begin{array}{cc} {\tilde{P}}_{Y|X}^{\left(s,v,b\right)}\left(y|x\right)\; & =\frac{P_{Y}\left(y\right)e^{{\tilde{v}}^{T}\left(y\right)s\left(x\right)+\tilde{d}\left(y\right)}}{\sum_{y^{\prime }\in Y}P_{Y}\left(y^{\prime }\right)e^{{\tilde{v}}^{T}\left(y^{\prime }\right)s\left(x\right)+\tilde{d}\left(y^{\prime }\right)}}, \end{array}$$

and the numerator can be written as

$$\begin{array}{cc} P_{Y}\left(y\right)e^{{\tilde{v}}^{T}\left(y\right)s\left(x\right)+\tilde{d}\left(y\right)}\; & =P_{Y}\left(y\right)\left(1+{\tilde{v}}^{T}\left(y\right)s\left(x\right)+\tilde{d}\left(y\right)+o\left(\epsilon \right)\right)\\ \; & =P_{Y}\left(y\right)\left(1+{\tilde{v}}^{T}\left(y\right)s\left(x\right)+\tilde{d}\left(y\right)\right)+o\left(\epsilon \right), \end{array}$$

where we have used (A39). Similarly, from

$$\begin{array}{cc} \sum_{y^{\prime }\in Y}P_{Y}\left(y\right)e^{{\tilde{v}}^{T}\left(y\right)s\left(x\right)+\tilde{d}\left(y\right)}\; & =\sum_{y^{\prime }\in Y}P_{Y}\left(y\right)\left(1+{\tilde{v}}^{T}\left(y\right)s\left(x\right)+\tilde{d}\left(y\right)\right)+o\left(\epsilon \right)\\ \; & =1+E_{P_{Y}}\left[{\tilde{v}}^{T}\left(Y\right)s\left(x\right)\right]+E_{P_{Y}}\left[\tilde{d}\left(y\right)\right]+o\left(\epsilon \right)\\ \; & =1+o(\epsilon ) \end{array}$$

we obtain

$$\frac{1}{\sum_{y^{\prime }\in Y}P_{Y}\left(y\right)e^{{\tilde{v}}^{T}\left(y\right)s\left(x\right)+\tilde{d}\left(y\right)}}=\frac{1}{1+o(\epsilon )}=1+o\left(\epsilon \right).$$

As a result, (A42) can be written as

(A43) $$\begin{array}{cc} {\tilde{P}}_{Y|X}^{\left(s,v,b\right)}\left(y|x\right)\; & =\left[P_{Y}\left(y\right)\left(1+{\tilde{v}}^{T}\left(y\right)s\left(x\right)+\tilde{d}\left(y\right)\right)+o\left(\epsilon \right)\right]\left[1+o\left(\epsilon \right)\right]\\ \; & =P_{Y}\left(y\right)\left(1+{\tilde{v}}^{T}\left(y\right)s\left(x\right)+\tilde{d}\left(y\right)\right)+o\left(\epsilon \right), \end{array}$$

which implies $P_{X}\;{\tilde{P}}_{Y|X}^{\left(v,b\right)}\in N_{C\epsilon }^{X\times Y}\left(P_{X}P_{Y}\right)$ for sufficiently small ϵ. In addition, the local assumption of distributions implies that $P_{XY}\in N_{\epsilon }^{X\times Y}\left(P_{X}P_{Y}\right)\subset N_{C\epsilon }^{X\times Y}\left(P_{X}P_{Y}\right)$. Again, from the local approximation of KL divergence [18]

(A44) $$D\left(P_{1}∥P_{2}\right)=\frac{1}{2}{∥\phi_{1}-\phi_{2}∥}^{2}+o\left(\epsilon^{2}\right),$$

we have

$$\begin{array}{cc} \; & D(P_{Y,X}∥P_{X}\;{\tilde{P}}_{Y|X}^{\left(s,v,b\right)})\\ \; & =\frac{1}{2}\sum_{x\in X,y\in Y}\frac{{\left[P_{Y,X}\left(y,x\right)-{\tilde{P}}_{Y|X}^{\left(s,v,b\right)}\left(y|x\right)P_{X}\left(x\right)\right]}^{2}}{P_{Y}\left(y\right)P_{X}\left(x\right)}+o\left(\epsilon^{2}\right)\\ \; & =\frac{1}{2}\sum_{x\in X,y\in Y}\left[\frac{P_{Y,X}\left(y,x\right)}{\sqrt{P_{Y}\left(y\right)P_{X}\left(x\right)}}-\sqrt{P_{Y}\left(y\right)P_{X}\left(x\right)}\right.\\ \; & \;{\left.-\sqrt{P_{Y}\left(y\right)P_{X}\left(x\right)}\left({\tilde{v}}^{T}\left(y\right)s\left(x\right)+\tilde{d}\left(y\right)+o\left(\epsilon \right)\right)\right]}^{2}+o\left(\epsilon^{2}\right)\\ \; & =\frac{1}{2}\sum_{x\in X,y\in Y}\left[\tilde{B}\left(y,x\right)-\sqrt{P_{Y}\left(y\right)P_{X}\left(x\right)}{\tilde{v}}^{T}\left(y\right)\tilde{s}\left(x\right)\right.\\ \; & \;\left.-\sqrt{P_{Y}\left(y\right)P_{X}\left(x\right)}\left(\tilde{d}\left(y\right)+\mu_{s}^{T}\tilde{v}\left(y\right)\right)\right.{\left.-\sqrt{P_{Y}\left(y\right)P_{X}\left(x\right)}o\left(\epsilon \right)\right]}^{2}+o\left(\epsilon^{2}\right)\\ \; & \overset{\left(*\right)}{=}\frac{1}{2}\sum_{x\in X,y\in Y}{\left[\tilde{B}\left(y,x\right)-\sqrt{P_{Y}\left(y\right)P_{X}\left(x\right)}{\tilde{v}}^{T}\left(y\right)\tilde{s}\left(x\right)\right]}^{2}\\ \; & \;+\frac{1}{2}\sum_{x\in X,y\in Y}{\left[\sqrt{P_{Y}\left(y\right)P_{X}\left(x\right)}\left(\tilde{d}\left(y\right)+\mu_{s}^{T}\tilde{v}\left(y\right)\right)\right]}^{2}+o\left(\epsilon^{2}\right)\\ \; & =\frac{1}{2}\sum_{x\in X,y\in Y}{\left[\tilde{B}\left(y,x\right)-{\left(\xi^{Y}\left(y\right)\right)}^{T}\xi^{X}\left(x\right)\right]}^{2}+\frac{1}{2}E_{P_{Y}}\left[{\left(\tilde{d}\left(y\right)+\mu_{s}^{T}\tilde{v}\left(y\right)\right)}^{2}\right]+o\left(\epsilon^{2}\right)\\ \; & =\frac{1}{2}{∥\tilde{B}-\Xi^{Y}{\left(\Xi^{X}\right)}^{T}∥}_{F}^{2}+\frac{1}{2}\eta^{\left(v,b\right)}\left(s\right)+o\left(\epsilon^{2}\right), \end{array}$$

where to obtain (*), we have used (A40) and (A41) together with the fact $|\tilde{B}\left(y,x\right)|<\epsilon$, and that

$$\begin{array}{c} \sum_{x\in X,y\in Y}\tilde{B}\left(y,x\right)\sqrt{P_{Y}\left(y\right)P_{X}\left(x\right)}\left(\tilde{d}\left(y\right)+\mu_{s}^{T}\tilde{v}\left(y\right)\right)=0,\\ \sum_{x\in X,y\in Y}P_{Y}\left(y\right)P_{X}\left(x\right){\tilde{v}}^{T}\left(y\right)\tilde{s}\left(x\right)\left(\tilde{d}\left(y\right)+\mu_{s}^{T}\tilde{v}\left(y\right)\right)=0, \end{array}$$

since $E\left[\tilde{d}\left(Y\right)\right]=0,E\left[\tilde{s}\left(X\right)\right]=E\left[\tilde{v}\left(Y\right)\right]=0$. □

## Appendix D. Proofs of Theorems 2 and 3

Theorems 2 and 3 can be proved based on Lemma 3.

**Proofs** **of** **Theorems** **2** **and** **3.** Note that the value of d(·) only affects the second term of the KL divergence; hence, we can always choose d(·) such that $\tilde{d}\left(y\right)+\mu_{s}^{T}\tilde{v}\left(y\right)=0$. Then, the $\left(\Xi^{Y},\Xi^{X}\right)$ pair should be chosen as

(A45) $${\left(\Xi^{Y},\Xi^{X}\right)}^{*}=\underset{\left(\Xi^{Y},\Xi^{X}\right)}{arg min}{∥\tilde{B}-\Xi^{Y}{\left(\Xi^{X}\right)}^{T}∥}_{F}^{2}.$$

Set the derivative (we use the denominator-layout notation of matrix calculus where the scalar-by-matrix derivative will have the same dimension as the matrix)

(A46) $$\frac{\partial }{\partial \Xi^{Y}}{∥\tilde{B}-\Xi^{Y}{\left(\Xi^{X}\right)}^{T}∥}_{F}^{2}=2\left(\Xi^{Y}{\left(\Xi^{X}\right)}^{T}\Xi^{X}-\tilde{B}\Xi^{X}\right)$$

to zero, and the optimal $\Xi^{Y}$ for fixed $\Xi^{X}$ is (here, we assume the matrix ${\left(\Xi^{X}\right)}^{T}\Xi^{X}=\Lambda_{\tilde{s}\left(X\right)}$ is invertible; for the case where ${\left(\Xi^{X}\right)}^{T}\Xi^{X}$ is singular, we can obtain a similar result with ordinary matrix inverse replaced by the Moore–Penrose inverse)

(A47) $$\Xi^{Y}{}^{*}=\tilde{B}\Xi^{X}{\left({\left(\Xi^{X}\right)}^{T}\Xi^{X}\right)}^{-1}.$$

As $1^{T}\sqrt{P_{Y}}\;\tilde{B}=0$, we have $1^{T}\sqrt{P_{Y}}\;\Xi^{Y}{}^{*}=0$, which demonstrates that $\Xi^{Y}{}^{*}$ is a valid matrix for a zero-mean feature vector. To express $\Xi^{Y}{}^{*}$ of (A47) in the form of *s* and *v*, we can make use of the correspondence between feature and information vectors. We can show that, for a zero-mean feature function f(X) with corresponding information vector ϕ, we have the correspondence $E_{P_{X|Y}}\left[f(X)|Y\right]↔\tilde{B}\phi$. To see this, note that the *y*-th element of information vector $\tilde{B}\phi$ is given by

$$\begin{array}{cc} \sum_{x\in X}\tilde{B}\left(y,x\right)\phi \left(x\right)\; & =\sum_{x\in X}\frac{P_{XY}\left(x,y\right)-P_{X}\left(x\right)P_{Y}\left(y\right)}{\sqrt{P_{X}\left(x\right)P_{Y}\left(y\right)}}f\left(x\right)\sqrt{P_{X}\left(x\right)}\\ \; & =\frac{1}{\sqrt{P_{Y}\left(y\right)}}\sum_{x\in X}P_{XY}\left(x,y\right)f\left(x\right)\\ \; & =\frac{1}{\sqrt{P_{Y}\left(y\right)}}E_{P_{X|Y}}\left[f(X)|Y=y\right]. \end{array}$$

Using similar methods, we can verify that $\Lambda_{\tilde{s}\left(X\right)}={\left(\Xi^{X}\right)}^{T}\Xi^{X}$. As a result, (A47) is equivalent to

(A48) $${\tilde{v}}^{*}\left(y\right)=E_{P_{X|Y}}\left[\Lambda_{\tilde{s}\left(X\right)}^{-1}\tilde{s}\left(X\right)|Y=y\right].$$

By a symmetry argument, we can also obtain the first two equations of Theorem 3. To obtain the third equations of these two theorems, we need to minimize $\eta^{\left(v,b\right)}\left(s\right)=E_{P_{Y}}\left[{\left(\mu_{s}^{T}\tilde{v}\left(Y\right)+\tilde{d}\left(Y\right)\right)}^{2}\right]$. For given $\tilde{v}$ and $\mu_{s}$, the optimal $\tilde{d}$ is

(A49) $${\tilde{d}}^{*}\left(y\right)=-\mu_{s}^{T}\tilde{v}\left(Y\right),$$

and the corresponding $\eta^{\left(v,b\right)}\left(s\right)=0$. In addition, for given $\tilde{d}$ and $\tilde{v}$, we have

(A50) $$\begin{array}{cc} \eta^{\left(v,b\right)}\left(s\right)\; & =E_{P_{Y}}\left[{\left(\mu_{s}^{T}\tilde{v}\left(Y\right)+\tilde{d}\left(Y\right)\right)}^{2}\right]\\ \; & =\mu_{s}^{T}\Lambda_{\tilde{v}\left(Y\right)}\;\mu_{s}+2\mu_{s}^{T}E_{P_{Y}}\left[\tilde{v}\left(Y\right)\tilde{d}\left(Y\right)\right]+\mathrm{var}\left(\tilde{d}\left(Y\right)\right). \end{array}$$

Set $\frac{\partial }{\partial \mu_{s}}\eta^{\left(v,b\right)}\left(s\right)=0$ and we obtain

(A51) $$\mu_{s}^{*}=-\Lambda_{\tilde{v}\left(Y\right)}^{-1}E_{P_{Y}}\left[\tilde{v}\left(Y\right)\tilde{d}\left(Y\right)\right].$$

□

## Appendix E. Proof of Theorem 4

**Proof.** From Lemma 3, choosing the optimal $\left(\Xi^{Y},\Xi^{X}\right)$ is equivalent to solving the matrix factorization problem of $\tilde{B}$. Since both $\Xi^{Y}$ and $\Xi^{X}$ have rank no greater than *k*, from the Eckart–Young–Mirsky theorem [56], the optimal choice of $\Xi^{Y}{\left(\Xi^{X}\right)}^{T}$ should be the truncated singular value decomposition of $\tilde{B}$ with top *k* singular values. As a result, ${\left(\Xi^{Y},\Xi^{X}\right)}^{*}$ are the left and right singular vectors of $\tilde{B}$ corresponding to the largest *k* singular values. The optimality of bias $\tilde{d}\left(y\right)=-\mu_{s}^{T}\tilde{v}\left(y\right)$ has already been shown in Appendix D. □

## Appendix F. Proof of Theorem 5

The following lemma is useful to prove Theorem 5.

**Lemma** **A6** (Pythagorean theorem). *Let $\Xi^{X}{}^{*}$ be the optimal matrix for given $\Xi^{Y}$ as defined in (13). Then,*

(A52) $$\begin{array}{c} {∥\tilde{B}-\Xi^{Y}{\left(\Xi^{X}\right)}^{T}∥}_{F}^{2}-{∥\tilde{B}-\Xi^{Y}{\left(\Xi^{X}{}^{*}\right)}^{T}∥}_{F}^{2}={∥\Xi^{Y}{\left(\Xi^{X}{}^{*}\right)}^{T}-\Xi^{Y}{\left(\Xi^{X}\right)}^{T}∥}_{F}^{2}. \end{array}$$

**Proof** **of** **Lemma** **A6.** Denote by 〈U,V〉 the Frobenius inner product of matrices U and V, i.e., $\langle U,V\rangle ≜\mathrm{tr}\left(U^{T}V\right)$, and we have

$$\begin{array}{cc} \langle \tilde{B}-\Xi^{Y}{\left(\Xi^{X}{}^{*}\right)}^{T},\Xi^{Y}{\left(\Xi^{X}\right)}^{T}\rangle \; & =\mathrm{tr}\left(\tilde{B}\Xi^{X}{\left(\Xi^{Y}\right)}^{T}\right)-\mathrm{tr}\left(\Xi^{X}{}^{*}{\left(\Xi^{Y}\right)}^{T}\Xi^{Y}{\left(\Xi^{X}\right)}^{T}\right)\\ \; & =\mathrm{tr}\left(\tilde{B}\Xi^{X}{\left(\Xi^{Y}\right)}^{T}\right)-\mathrm{tr}\left({\tilde{B}}^{T}\Xi^{Y}{\left(\Xi^{X}\right)}^{T}\right)\\ \; & =0. \end{array}$$

As a result, we obtain

$$\begin{array}{cc} {∥\tilde{B}-\Xi^{Y}{\left(\Xi^{X}\right)}^{T}∥}_{F}^{2}\; & ={∥\tilde{B}-\Xi^{Y}{\left(\Xi^{X}{}^{*}\right)}^{T}+\left(\Xi^{Y}{\left(\Xi^{X}{}^{*}\right)}^{T}-\Xi^{Y}{\left(\Xi^{X}\right)}^{T}\right)∥}_{F}^{2}\\ \; & ={∥\tilde{B}-\Xi^{Y}{\left(\Xi^{X}{}^{*}\right)}^{T}∥}_{F}+{∥\Xi^{Y}{\left(\Xi^{X}{}^{*}\right)}^{T}-\Xi^{Y}{\left(\Xi^{X}\right)}^{T}∥}_{F}^{2}\\ \; & \;+2\langle \tilde{B}-\Xi^{Y}{\left(\Xi^{X}{}^{*}\right)}^{T},\Xi^{Y}\left({\left(\Xi^{X}{}^{*}\right)}^{T}-{\left(\Xi^{X}\right)}^{T}\right)\rangle \\ \; & ={∥\tilde{B}-\Xi^{Y}{\left(\Xi^{X}{}^{*}\right)}^{T}∥}_{F}+{∥\Xi^{Y}{\left(\Xi^{X}{}^{*}\right)}^{T}-\Xi^{Y}{\left(\Xi^{X}\right)}^{T}∥}_{F}^{2}, \end{array}$$

which finishes the proof. □

Proceeding to our proof of Theorem 5, from Lemma A6 we have

$$\begin{array}{cc} \; & L\left(s\right)-L\left(s^{*}\right)\\ \; & \;=\frac{1}{2}\left[∥\tilde{B}-\Xi^{Y}{\left(\Xi^{X}\right)}^{T}{∥}_{F}^{2}-{∥\tilde{B}-\Xi^{Y}{\left(\Xi^{X}{}^{*}\right)}^{T}∥}_{F}^{2}\right]+\frac{1}{2}\left[\eta^{\left(v,b\right)}\left(s\right)-\eta^{\left(v,b\right)}\left(s^{*}\right)\right]+o\left(\epsilon^{2}\right)\\ \; & \;=\frac{1}{2}{∥\Xi^{Y}{\left(\Xi^{X}{}^{*}\right)}^{T}-\Xi^{Y}{\left(\Xi^{X}\right)}^{T}∥}_{F}^{2}+\frac{1}{2}\kappa^{\left(v,b\right)}\left(s,s^{*}\right)+o\left(\epsilon^{2}\right), \end{array}$$

where $\kappa^{\left(v,b\right)}\left(s,s^{*}\right)≜\eta^{\left(v,b\right)}\left(s\right)-\eta^{\left(v,b\right)}\left(s^{*}\right)$. We then optimize $∥\Xi^{Y}{\left(\Xi^{X}{}^{*}\right)}^{T}-\Xi^{Y}{\left(\Xi^{X}\right)}^{T}{∥}_{F}^{2}$ and $\kappa^{\left(v,b\right)}\left(s,s^{*}\right)$ separately.

For the first term, we need to express $\Xi^{X}$ in terms of W and $\Xi_{1}^{X}$. From (17), we obtain

(A53) $$\begin{array}{c} E\left[s_{z}\left(X\right)\right]=\sigma \left(c(z)\right)+o\left(\epsilon \right), \end{array}$$

(A54) $$\begin{array}{c} {\tilde{s}}_{z}\left(x\right)=w^{T}\left(z\right)\tilde{t}\left(x\right)\cdot \sigma^{\prime }\left(c(z)\right)+o\left(\epsilon \right), \end{array}$$

which can be expressed in information vectors as

(A55) $$\Xi^{X}=\Xi_{1}^{X}W^{T}J+o\left(\epsilon \right).$$

From Theorem 3, we have

(A56) $$\Xi^{X}{}^{*}={\tilde{B}}^{T}\;\Xi^{Y}\;{\left({\left(\Xi^{Y}\right)}^{T}\Xi^{Y}\right)}^{-1}.$$

As a result, we have

(A57) $$\begin{array}{cc} {∥\Xi^{Y}{\left(\Xi^{X}{}^{*}\right)}^{T}-\Xi^{Y}{\left(\Xi^{X}\right)}^{T}∥}_{F}^{2}\; & ={∥{\left({\left(\Xi^{Y}\right)}^{T}\Xi^{Y}\right)}^{1/2}\left({\left(\Xi^{X}{}^{*}\right)}^{T}-{\left(\Xi^{X}\right)}^{T}\right)∥}_{F}^{2}\\ \; & =∥{\left({\left(\Xi^{Y}\right)}^{T}\Xi^{Y}\right)}^{1/2}\cdot \left({\left(\Xi^{X}{}^{*}\right)}^{T}-JW{\left(\Xi_{1}^{X}\right)}^{T}-o\left(\epsilon \right)\right){∥}_{F}^{2}\\ \; & =∥{\left({\left(\Xi^{Y}\right)}^{T}\Xi^{Y}\right)}^{1/2}\cdot \left({\left(\Xi^{X}{}^{*}\right)}^{T}-JW{\left(\Xi_{1}^{X}\right)}^{T}\right){∥}_{F}^{2}+o\left(\epsilon^{2}\right)\\ \; & =∥{\left({\left(\Xi^{Y}\right)}^{T}\Xi^{Y}\right)}^{1/2}J\cdot \left(J^{-1}{\left(\Xi^{X}{}^{*}\right)}^{T}-W{\left(\Xi_{1}^{X}\right)}^{T}\right){∥}_{F}^{2}+o\left(\epsilon^{2}\right)\\ \; & ={∥\Theta {\tilde{B}}_{1}-\Theta W{\left(\Xi_{1}^{X}\right)}^{T}∥}_{F}^{2}+o\left(\epsilon^{2}\right), \end{array}$$

where the third equality follows from the fact that [cf. (A41)] $\tilde{s}\left(x\right)=O\left(\epsilon \right)$ and $\tilde{v}\left(y\right)=O\left(1\right)$, and the last equality follows from the definitions ${\tilde{B}}_{1}≜J^{-1}{\left(\Xi^{X}{}^{*}\right)}^{T}$ and $\Theta ≜{\left({\left(\Xi^{Y}\right)}^{T}\Xi^{Y}\right)}^{1/2}J$.

For the second term, from (A50) and (A51), we have

(A58) $$\begin{array}{cc} \kappa^{\left(v,b\right)}\left(s,s^{*}\right)\; & ={\left[\left(\mu_{s}-\mu_{s^{*}}\right)+\mu_{s^{*}}\right]}^{T}\Lambda_{\tilde{v}\left(Y\right)}\left[\left(\mu_{s}-\mu_{s^{*}}\right)+\mu_{s^{*}}\right]\\ \; & \;-\mu_{s^{*}}^{T}\Lambda_{\tilde{v}\left(Y\right)}\mu_{s^{*}}+2{\left(\mu_{s}-\mu_{s^{*}}\right)}^{T}E_{P_{Y}}\left[\tilde{v}\left(Y\right)\tilde{d}\left(Y\right)\right]\\ \; & ={\left(\mu_{s}-\mu_{s^{*}}\right)}^{T}\Lambda_{\tilde{v}\left(Y\right)}\left(\mu_{s}-\mu_{s^{*}}\right)+2{\left(\mu_{s}-\mu_{s^{*}}\right)}^{T}\left(\Lambda_{\tilde{v}\left(Y\right)}\mu_{s^{*}}+E_{P_{Y}}\left[\tilde{v}\left(Y\right)\tilde{d}\left(Y\right)\right]\right)\\ \; & ={\left(\mu_{s}-\mu_{s^{*}}\right)}^{T}\Lambda_{\tilde{v}\left(Y\right)}\left(\mu_{s}-\mu_{s^{*}}\right). \end{array}$$

Combining (A57) and (A58) finishes the proof.

## Appendix G. Analyses of Hidden Layer Parameters

First, from (A53), the bias c(z) of hidden layer is (when $\mu_{t}\neq 0$, the formula should be modified as $c\left(z\right)=\sigma^{-1}\left(\mu_{s}^{*}\left(z\right)\right)-\mu_{t}^{T}w+o\left(\epsilon \right)$.)

$$c\left(z\right)=\sigma^{-1}\left(\mu_{s}^{*}\left(z\right)\right)+o\left(\epsilon \right).$$

To obtain $\mu_{s}^{*}$, let us define $\sigma_{\min}≜\inf_{x}\sigma \left(x\right),\sigma_{\max}≜\sup_{x}\sigma \left(x\right)$. Then, the optimal $\mu_{s}$ is the solution of

(A59) $$\begin{array}{cccc} \; & \underset{\mu_{s}}{\mathrm{minimize}} & \; & {\left(\mu_{s}-\mu_{s^{*}}\right)}^{T}\Lambda_{\tilde{v}\left(Y\right)}\left(\mu_{s}-\mu_{s^{*}}\right)\\ \; & \mathrm{subject}\;\mathrm{to} & \; & \sigma_{\min}⪯\mu_{s}⪯\sigma_{\max}. \end{array}$$

If $\mu_{s^{*}}$ satisfies the constraint of (A59), then it is the optimal solution. Otherwise, some elements of $\mu_{s}^{*}$ will become either $\sigma_{\min}$ or $\sigma_{\max}$, known as the saturation phenomenon [21].

To obtain $W{}^{*}$, let

$$\begin{array}{c} {\tilde{B}}_{1}^{\prime }≜\Theta {\tilde{B}}_{1}={\left({\left(\Xi^{Y}\right)}^{T}\Xi^{Y}\right)}^{-1/2}\;{\left(\Xi^{Y}\right)}^{T}\;\tilde{B},\\ W{}^{\prime }≜\Theta W={\left({\left(\Xi^{Y}\right)}^{T}\Xi^{Y}\right)}^{1/2}JW. \end{array}$$

Then, the optimal $W{}^{\prime }$ is given by

(A60) $$W^{\prime *}=\underset{W{}^{\prime }}{arg min}\;{∥{\tilde{B}}_{1}{}^{\prime }-W{}^{\prime }{\left(\Xi_{1}^{X}\right)}^{T}∥}_{F}^{2}={\tilde{B}}_{1}{}^{\prime }\Xi_{1}^{X}{\left({\left(\Xi_{1}^{X}\right)}^{T}\Xi_{1}^{X}\right)}^{-1}.$$

Hence, $W{}^{*}$ is given by

$$\begin{array}{cc} W{}^{*}=\Theta^{-1}W^{\prime *}\; & =\Theta^{-1}{\tilde{B}}_{1}^{\prime }\Xi_{1}^{X}{\left({\left(\Xi_{1}^{X}\right)}^{T}\Xi_{1}^{X}\right)}^{-1}\\ \; & ={\tilde{B}}_{1}\Xi_{1}^{X}{\left({\left(\Xi_{1}^{X}\right)}^{T}\Xi_{1}^{X}\right)}^{-1}\\ \; & =J^{-1}\cdot {\left[\Xi^{Y}{\left({\left(\Xi^{Y}\right)}^{T}\Xi^{Y}\right)}^{-1}\right]}^{T}\tilde{B}\;\Xi_{1}^{X}{\left({\left(\Xi_{1}^{X}\right)}^{T}\Xi_{1}^{X}\right)}^{-1}, \end{array}$$

where the term $\tilde{B}\;\Xi_{1}^{X}{\left({\left(\Xi_{1}^{X}\right)}^{T}\Xi_{1}^{X}\right)}^{-1}$ corresponds to a feature projection of $\tilde{t}\left(X\right)$:

(A61) $$\tilde{B}\;\Xi_{1}^{X}\;{\left({\left(\Xi_{1}^{X}\right)}^{T}\Xi_{1}^{X}\right)}^{-1}↔E_{P_{X|Y}}\left[\Lambda_{\tilde{t}\left(X\right)}^{-1}\tilde{t}\left(X\right)|Y\right].$$

As a consequence, this multi-layer neural network conducts a generalized feature projection between features extracted from different layers. Note that the projected feature $E_{P_{\tilde{t}|Y}}\left[\Lambda_{\tilde{t}}^{-1}\tilde{t}|Y\right]$ depends only on the distribution $P_{\tilde{t}|Y}$ and does not depend on the distribution $P_{X|Y}$. Therefore, the above computations can be accomplished without knowing the hidden random variable *X* and can be applied to general cases.
